# Benzo[*d*]thiazole-2-thiol bearing 2-oxo-2-substituted-phenylethan-1-yl as potent selective *lasB* quorum sensing inhibitors of Gram-negative bacteria†

**DOI:** 10.1039/d1ra03616e

**Published:** 2021-08-26

**Authors:** Tung Truong Thanh, Huy Luong Xuan, Thang Nguyen Quoc

**Affiliations:** PHENIKAA University Hanoi 12116 Vietnam tung.truongthanh@phenikaa-uni.edu.vn http://tunglab.com; PHENIKAA Institute for Advanced Study (PIAS), PHENIKAA University Hanoi 12116 Vietnam; Nuclear Medicine Unit, Vinmec Healthcare System Hanoi 10000 Vietnam

## Abstract

Quorum sensing is a well-known term for describing bacterial cell–cell communication. Bacteria use quorum sensing pathways to respond to external factors such as nutrient availability, defense mechanisms, and coordinate host toxic behaviors such as biofilm formation, virulence production, and other pathogenesis. Discovery of novel compounds which inhibit quorum sensing without being antibiotic are currently emerging fields. Herein, the library of fifteen benzo[*d*]thiazole/quinoline-2-thiol bearing 2-oxo-2-substituted-phenylethan-1-yl compounds was designed, synthesized and evaluated to find novel quorum sensing inhibitors. Firstly, compounds were evaluated for their growth inhibitory activities at high concentrations up to 1000 μg mL^−1^ toward *Pseudomonas aeruginosa*. Under our conditions, twelve compounds showed moderate growth inhibitory activities in the concentration tested. To our delight, three compounds 3, 6 and 7 do not affect the growth of the bacteria which were chosen for the evaluation of quorum sensing inhibitor activities. In the *LasB* system, our compounds 3, 6, 7 showed promising quorum-sensing inhibitors with IC_50_ of 115.2 μg mL^−1^, 182.2 μg mL^−1^ and 45.5 μg mL^−1^, respectively. In the *PqsR* system, no activity observed suggesting that the selectivity of the compound toward the *LasB* system. In addition, 7 showed the moderate anti-biofilm formation of *Pseudomonas aeruginosa*. Docking studies revealed that 3, 6 and 7 binding to the active site of *Pseudomonas aeruginosa* quorum sensing *LasR* system with better affinity compared to reference compounds 4-NPO. Finally, computation calculations suggest that compounds are a good template for further drug development.

## Abstract 2

Benzo[*d*]thiazole-2-thiol bearing 2-oxo-2-substituted-phenylethan-1-yl as potent selective *lasB* quorum sensing inhibitors and anti-biofilm formation of *Pseudomonas aeruginosa*.

## Graphical abstract

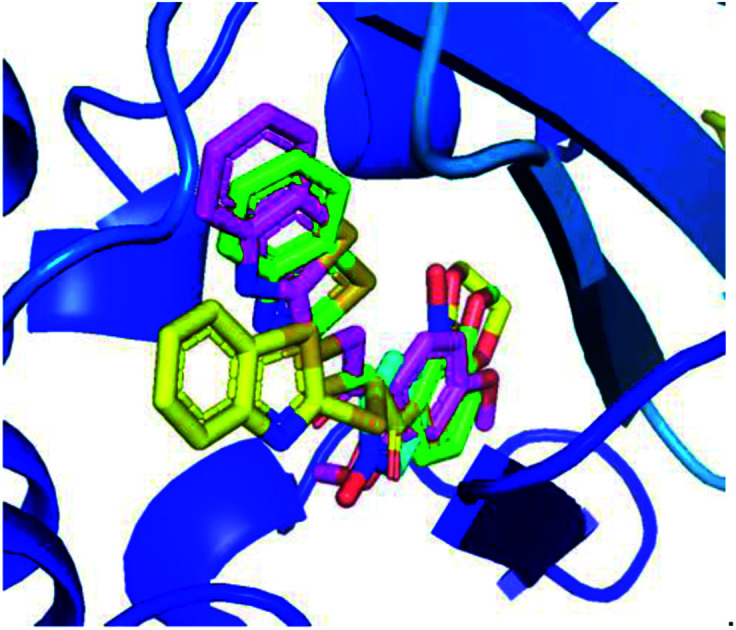

## Introduction

Quorum sensing (QS) is a term used for the phenomena where bacteria use small molecules termed signal molecules or autoinducers for cell–cell communication, responses to external factors such as nutrient availability, defense mechanisms as well as to coordinate behavior such as biofilm formation and pathogenesis.^1–3^ QS is a challenging new target for the development of new drugs for antimicrobial treatments. The studies on QS are conducted to modulates either signal molecule responses or alternating signal molecule supply. The QS inhibitors (QSI) are thereby jamming interbacterial communication and organization rather than being cytotoxic.^1,2^ It is hypothesized that such QS based antibiotic agents are less prone to selection for resistance than traditional bacteriostatic or bactericidal remedies.^1–3^

**Fig. 1:**
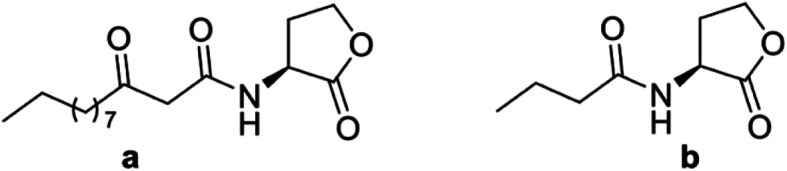
Signal molecules *N*-(3-oxododecanoyl)-l-homoserine lactone (a) and *N*-butanoyl-l-homoserine lactone (b).

In Gram-negative bacteria, the QS signal compounds are *N*-acyl-l-homoserine lactones (AHLs, Fig. 1).^4–12^ Using AHLs, bacteria can promote the gene expression process in connection with detection of population density.^7–10^ Therefore, compounds having QS inhibitory activity but void of antibiotic activity are potent agents in preventing the formation of biofilm, reducing the production of toxins, and, most importantly, discourage bacteria to develop future resistance.^7–13^ Many QS inhibitors have been reported which based on the structure of AHLs.^4,5,12,13^

Several of QSIs are developed as a single antivirulence therapy.^4*a*,*b*^ For example, RIP is a potent agent for the treatment of *Staphylococcus aureus* biofilm;^4*c*^ In the *Caenorhabditis elegans* infection model, QSI compounds 4-NPO or garlic extract prevents the toxicity of *Pseudomonas aeruginosa* toward *Caenorhabditis elegans*;^4*d*^ In an animal model, treatment of the *Pseudomonas aeruginosa* infected mice with halogenated furanones results in increasing survival rate and eliminating the bacteria faster.^4*e*,*f*^ In clinical, the combination of QSIs and antibiotics are currently the most effective way. For example, the addition of ajoene enhances the activity of the antibiotic tobramycin;^5*a*–*c*^ The combination use of gallocatechin 3-gallate enhances the activity of tetracycline;^5*d*^ The resistance of *pseudomonas aeruginosa* biofilm towards antibiotics tobramycin and ciprofloxacin can be reverted by QSI compound *N*-(2-pyrimidyl) butylamine.^5*e*^ Moreover, several of QSI compounds are in clinical trials such as garlic for the treatment of cystic fibrosis patients;^5*f*^ anticancer and QSI active compound fluorouracil for the prevention of catheter-associated infection.^5*g*,*h*^ Noteworthy, the antibiotic drug azithromycin can be used to treat cystic fibrosis at the non-antibiotic concentration due to its QSI activity.^5*i*^

**Fig. 2:**
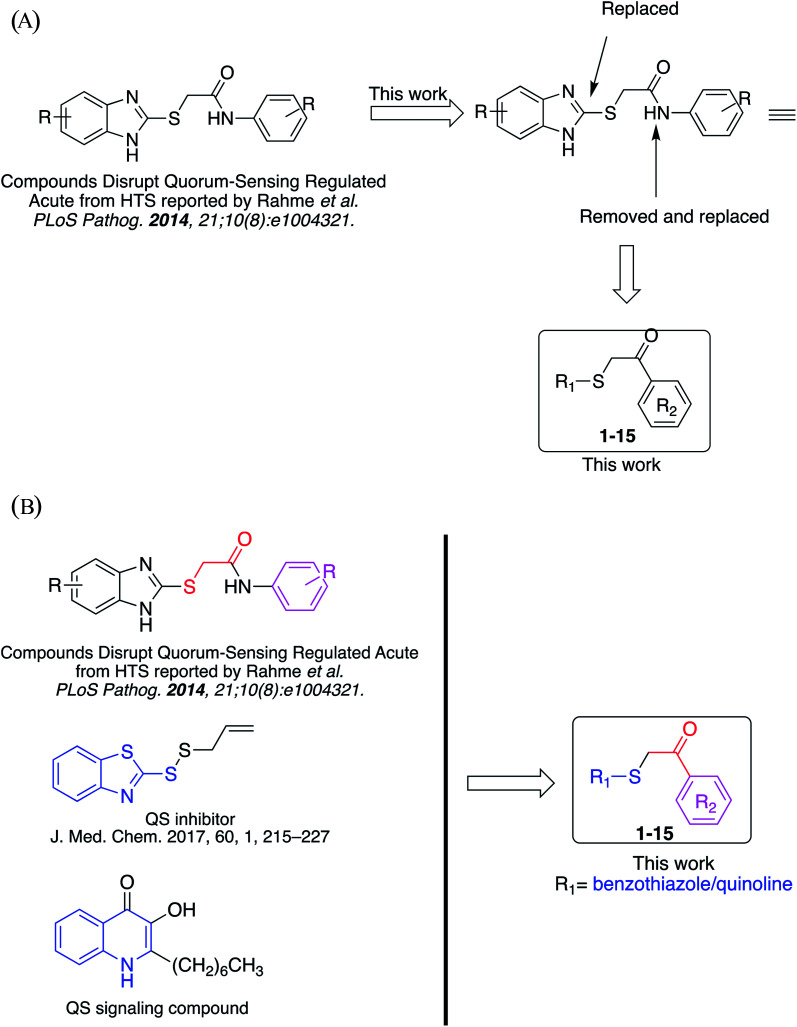
(A) The studies conducted in this work. (B) Design of library compounds.

Rahme *et al.* at Harvard University have reported the series of 2-((substituted-benzo[*d*]imidazol-2-yl)thio)-*N*-substituted-acetamide that interfere with quorum-sensing activities from high-throughput whole-cell screening (Fig. 2A).^14*a*^ However, in terms of medicinal chemistry research, none of the synthesis and rational studies investigating benzo[*d*]thiazole/quinoline-2-thiol skeleton as novel QS inhibitors are reported to date.

Herein, we have designed, synthesized and evaluated a library of novel benzo[*d*]thiazole/quinoline-2-thiol bearing 2-oxo-2-substituted-phenylethan-1-yl compounds targeting *LasB* QS system which inspired from these initial observations. In this study, fifteen compounds will be synthesized and evaluated for their antimicrobial activities and QS inhibitory activites in *P. aeruginosa* QS reporter strains *lasB-gfp*. In addition, docking and computation prediction for drug-likeness of the compounds will also be performed.

## Results and discussion

### Design of library compounds

Previously, benzothiazole-containing compounds were reported as non-selective QS inhibitors from our collaborators.^14*b*^ In addition, as mentioned earlier, from high-throughput whole-cell screening the series of compounds containing 1-yl-thiopropan-2-one bridge that interfere with quorum-sensing activities were reported (Fig. 2A).^14*a*^ In this work, we have designed the compounds containing benzo[*d*]thiazole-2-thiol moiety conjugated substituted-phenyl *via* 1-yl-thiopropan-2-one compounds. Furthermore, quinoline as an important moiety of *Pseudomonas* Quinolone Signal (PQS) was also designed as rational for benzo[*d*]thiazole^14*c*,19^ (Fig. 2B). The synthesis of the library compounds was described as follow.

#### Synthesis of benzo[*d*]thiazole-2-thiol bearing 2-oxo-2-substituted-phenylethan-1-yl

**Scheme 1:**
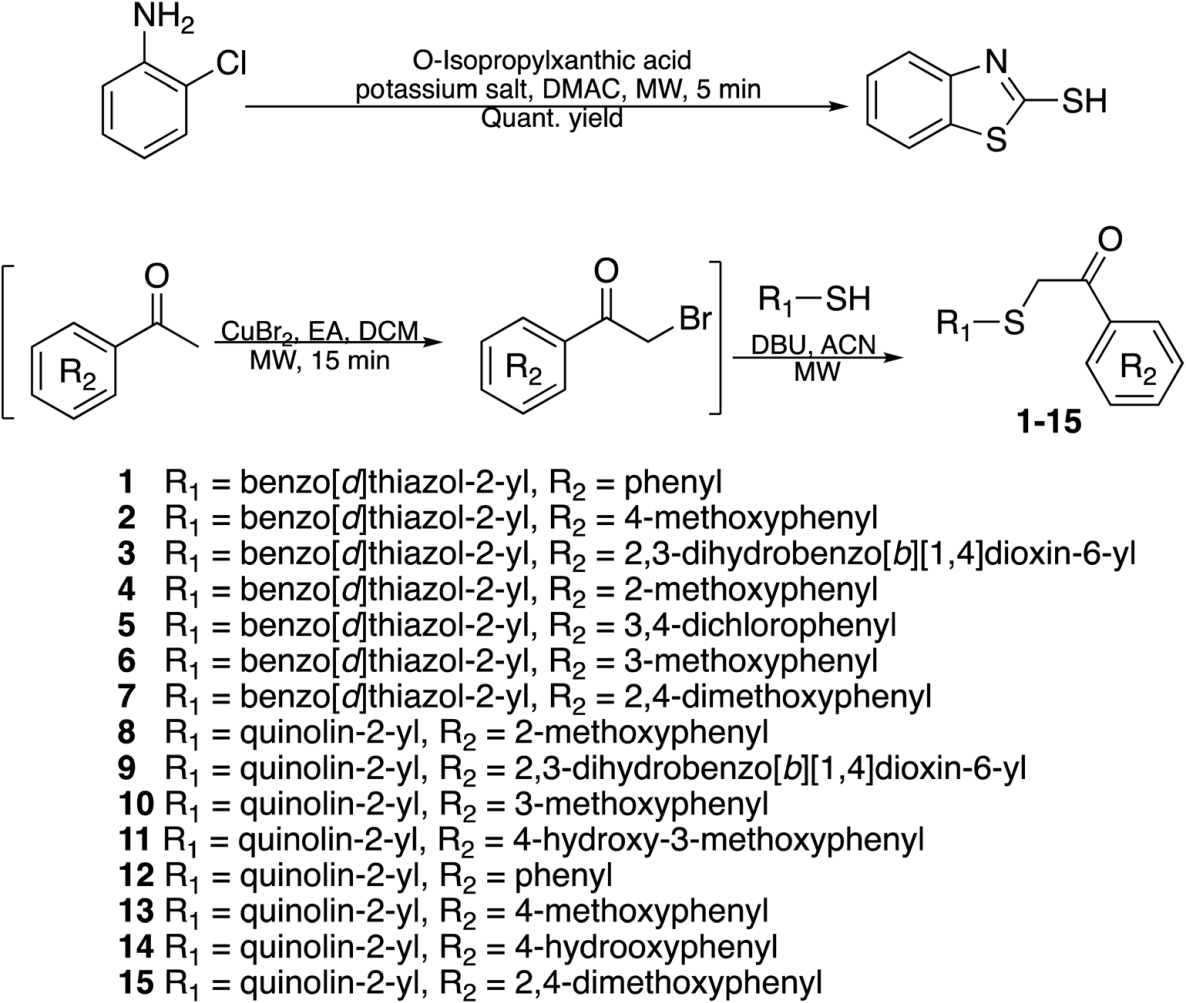
Synthetic scheme for the preparation of the library compounds.

The synthesis of compound libraries was started from the preparation of benzo[*d*]thiazole-2-thiol from *o*-haloanilines using the new synthetic method reported previously from our group.^22^ Then benzo[*d*]thiazole-2-thiol reacts with corresponding 2-bromo-1-substituted-phenylethan-1-one (which was prepared from the corresponding ketone). The reactions were performed under microwave irradiation to afford final products 1–6 with excellent yields (Scheme 1).

#### Synthesis of quinoline-containing compounds

Quinoline-2-thiol reacts with corresponding 2-bromo-1-substituted-phenylethan-1-one (which was prepared from the corresponding ketone) to afford products with excellent yields (Scheme 1).

In this study, fifteen benzo[*d*]thiazole/quinoline-2-thiol bearing 2-oxo-2-substituted-phenylethan-1-yl compounds were designed and synthesized for possible QS inhibitors. Only interesting compounds which do not affect the growth of bacteria will be selected for QS evaluation. Therefore, the synthesized compounds were firstly screening the antimicrobial activities toward *P. aeruginosa*. The results were summarized in Table 1.

**Table 1:** Growth inhibitory activities of the synthesized compounds^a^

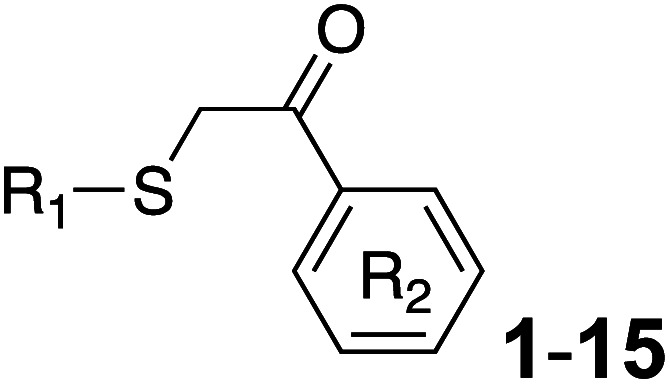				
Cpds	R_1_	R_2_	MW	MIC range (μg mL^−1^)
1	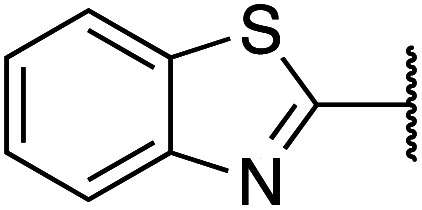	Phenyl	285	<128
2	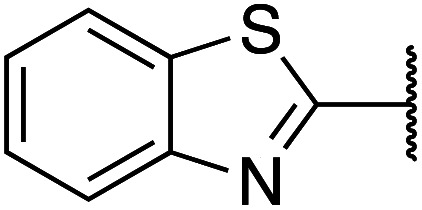	4-Methoxyphenyl	315	<256
3	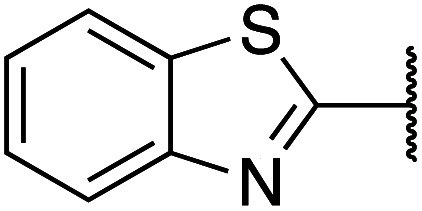	2,3-Dihydrobenzo[*b*][1,4]dioxin-6-yl	343	>512
4	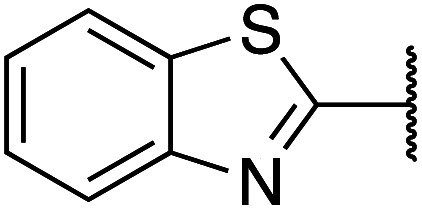	2-Methoxyphenyl	315	<256
5	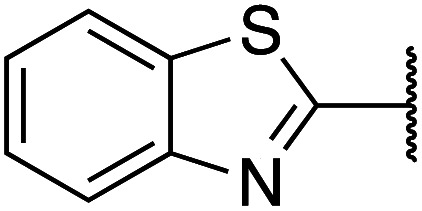	3,4-Dichlorophenyl	354	<128
6	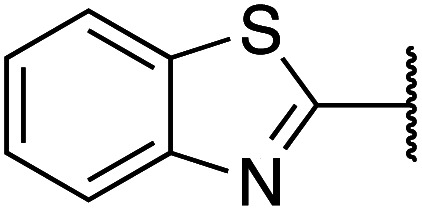	3-Methoxyphenyl	315	>512
7	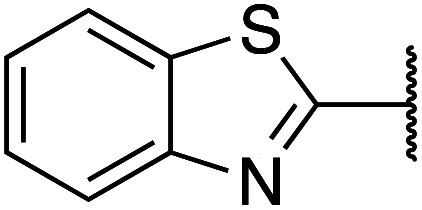	2,4-Dimethoxyphenyl	345	>512
8	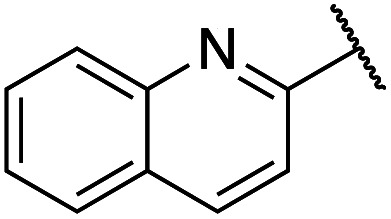	2-Methoxyphenyl	309	<128
9	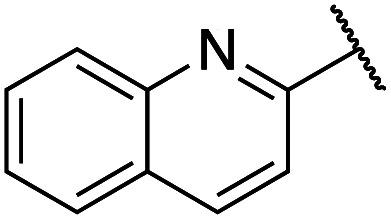	2,3-Dihydrobenzo[*b*][1,4]dioxin-6-yl	337	<128
10	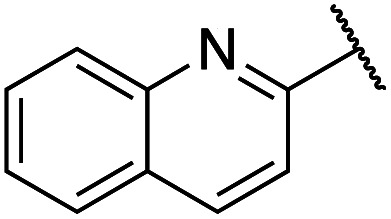	3-Methoxyphenyl	309	<256
11	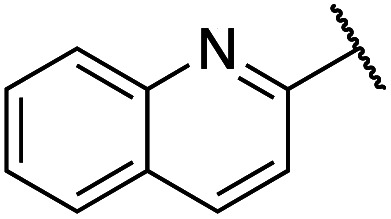	4-Hydroxy-3-methoxyphenyl	325	<128
12	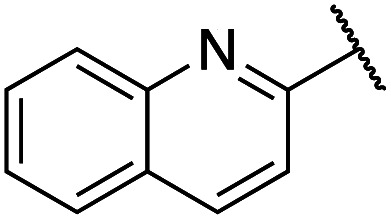	Phenyl	279	<128
13	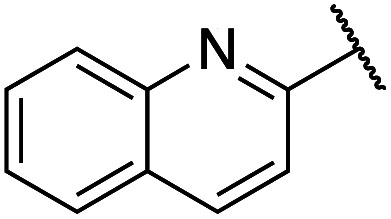	4-Methoxyphenyl	309	<256
14	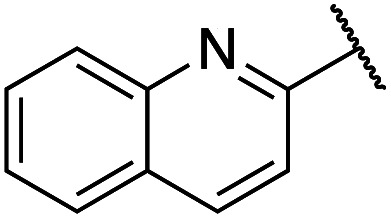	4-Hydrooxyphenyl	295	<64
15	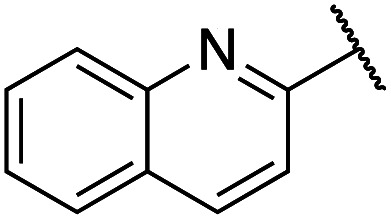	2,4-Dimethoxyphenyl	339	<128
Ref	Imipenem^b^			0.15

a Experiments were performed in triplicate.

b Positive control.

As showed in Table 1, compounds 1–2, 4–5, 8–15 exhibited some inhibitory activities toward *P. aeruginosa* with MIC ranges less than 64 μg mL^−1^ (14), 128 μg mL^−1^ (1, 5, 8, 9, 11, 12, 15), and 256 μg mL^−1^ (2, 4, 10, 13). Only 3, 6, and 7 do not affect the growth of the bacteria at the concentrations tested. In term of antimicrobial activities, our data reveals that the presence of *O*-alkyl moiety at position 2, 3 and 4 of the phenyl group eliminates the antimicrobial activity of the benzo[*d*]thiazole compounds in this series (compounds 3, 6, 7). Since QS promising compound is required that they are not interfering with the growth of bacteria.^1–12^ In this work, 3, 6, and 7 were selected for the QS evaluation as the next step. The study on antimicrobial activities of the rest compounds (MIC < 128 μg mL^−1^, Table 1) will be investigated in our other project.

**Fig. 3:**
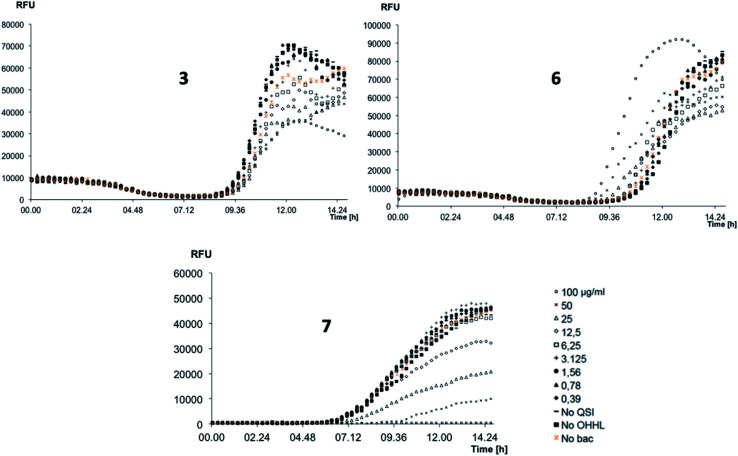
QS inhibitory activities of compounds 3, 6, 7 screened with the *lasB-gfp* (*P. aeruginosa*) monitor strain. GFP fluorescence (RFU), GFP expression is controlled by the QS controlled lasB promoter. Compounds were tested in triplicate and in 2-fold dilutions from 100 μg mL^−1^. No QSI: control with no addition of test compound. No OHHL: *N*-(3-oxohexanoyl)-l-homoserine lactone added. No bac: no bacteria.

The *P. aeruginosa* QS reporter strains *lasB-gfp* was used for screening the inhibition of QS system for 3, 6, and 7.^17^ The results were depicted in Fig. 3.

To our delight, 3, 6, and 7 showed very promising QS inhibitory activities within the concentration ranges tested. Compound 7 reduced QS activity at concentration around 12.5 μg mL^−1^ whereas 3 and 6 showed activities around 100 μg mL^−1^. The quantitative evaluation of QS inhibitory activities was summarized in Table 2.

**Table 2:** IC_50_ of compounds toward *lasB-gfp* (*P. aeruginosa*) monitor strain

Compounds	IC_50_ (QSI), μg mL^−1^
3	115.2 ± 1.32
6	182.2 ± 2.20
7	45.5 ± 1.05
4-NPO^a^	15.4 ± 2.44

a QSI-reference 4-nitropyridine-*N*-oxide.^23^

As a result, compound 7 is the strongest QS inhibitor with IC_50_ of 45.5 μg mL^−1^. 3 and 6 showed similar activity with IC_50_ of 115.2 μg mL^−1^ and 182.2 μg mL^−1^, respectively. Under our testing condition, reference compound 4-NPO showed QSI at 15.4 μg mL^−1^.^23^ It should be noted that, no QSI activities of the compounds observed in *PqsR-Pseudomonas* system (data not shown). Those results suggest that our compounds selectivity inhibit the *LasB* over *PqsR QS* system.

### Inhibition of bacterial biofilm formation

**Fig. 4:**
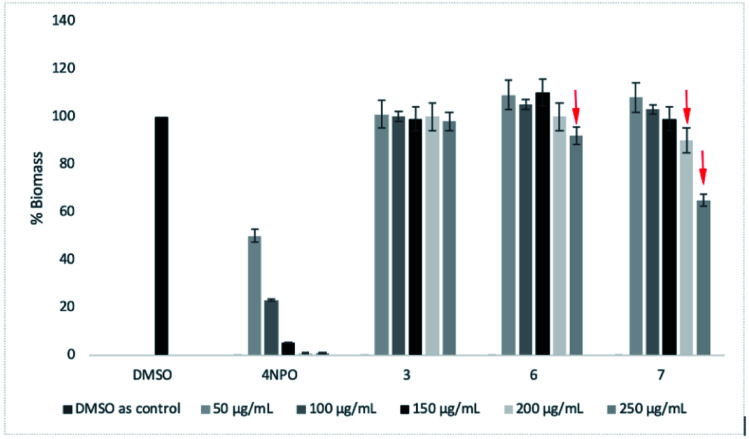
Quantification of the biofilm formation results of 4-NPO, 3, 6, and 7 toward *P. aeruginosa* after 48 h growth with the concentration ranges from 50 μg mL^−1^ to 250 μg mL^−1^. DMSO is negative control. The error bars show the SD of anti-biofilm formation from three independent assays.

Next, three most active compounds 3, 6 and 7 were selected for evaluation of anti-biofilm formation along with reference compound 4-NPO. The *P. aeruginosa* PA14 was used for the assay using the method as previously described^24,25^ with slightly modification. Compounds was test with the concentrations of 50 μg mL^−1^, 100 μg mL^−1^, 150 μg mL^−1^, 200 μg mL^−1^ and 250 μg mL^−1^. DMSO was used as negative control. 4-NPO was used as positive control. Firstly, the compounds were tested the inhibitory activity at highest concentration of up to 400 μg mL^−1^ for each compound. To our delight, 3, 6 and 7 do not show bactericidal or bacteriostatic activities in comparison to DMSO within the concentration tested (data not shown). Then, *P. aeruginosa* PA14 was treated with respective 50 μg mL^−1^, 100 μg mL^−1^, 150 μg mL^−1^, 200 μg mL^−1^ and 250 μg mL^−1^ of each compounds and incubation for 48 h. The calculation of total Biofilm mass was depicted in Fig. 4 as previously described.^24^

As a result, compound 7 showed moderate anti-biofilm activity by reducing 40% of total biomass (Fig. 4). Compound 3 does not show any inhibitory activities of concentration up to 250 μg mL^−1^. A slightly reducing of biomass was seen for compound 6 at 250 μg mL^−1^. Under our condition, 4-NPO showed strong anti-biofilm formation by gradually reducing the total biomass within concentration tested. 4-NPO totally inhibit the formation of biofilm at concentration of above 150 μg mL^−1^.

**Fig. 5:**
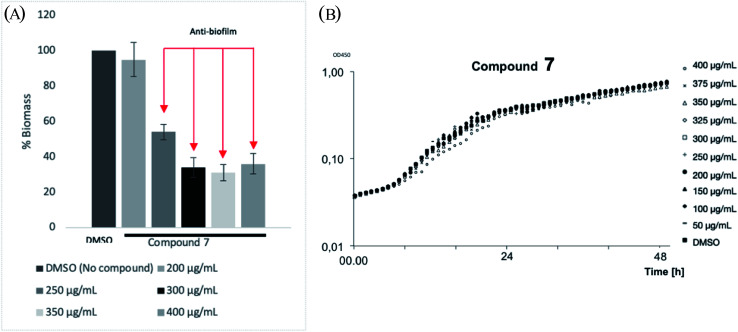
(A) Quantification of the biofilm formation results of 7 toward *P. aeruginosa* after 48 h growth with the concentration ranges from 200 μg mL^−1^ to 400 μg mL^−1^. DMSO is negative control. The error bars show the SD of anti-biofilm formation from three independent assays. (B) Bacterial growth (OD_450_, *P. aeruginosa*) treatment with difference concentrations of 7.

To understand the level of anti-biofilm formation of 7, higher concentrations of this compound were applied (Fig. 5A). Consequently, 7 showed clearly the anti-biofilm formation of *P. aeruginosa* at concentrations from 250 μg mL^−1^. The maximum inhibition of bio-film biomass was observed as 70% at 300 μg mL^−1^ of 7. No bactericidal or bacteriostatic activities of 7 were observed within concentrations tested (Fig. 5B). Even though a high concentration of 7 is required for antibiofilm activities, making compounds unsuitable for oral administration, it could still be beneficial for use as topical administration. Those results suggest that compound 7 is a promising template for further drug development.

It should be noted that the activities of our hit compounds toward the HSL-mediated QS system in *Chromobacterium violaceum* (anti-violacein activities) were also performed. However, no significant activities were observed (data not shown).

#### The cytotoxicity for 3, 6, 7

**Fig. 6:**
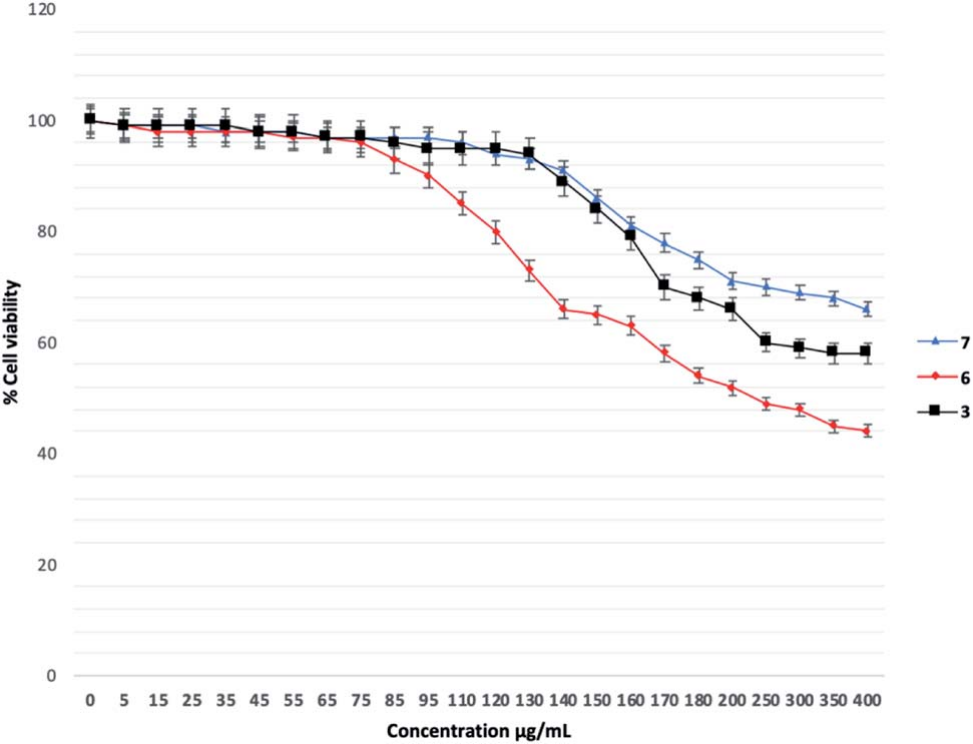
The cytotoxicity of compounds 3, 6, 7.

The cytotoxicity assay was performed using the MTT method for the hit compounds 3, 6, 7 to better understand the druggability in humans. The compounds were tested with concentrations from 1 μg mL^−1^ to 400 μg mL^−1^. To our delight, all compounds posed moderate toxicity against HeLa cells as evidenced by the high percentage of cell viability (around 60%) at the QSI concentrations of less than 200 μg mL^−1^ (as depicted in Fig. 6). Compound 6 showed significant cytotoxicity at the highest concentration of 400 μg mL^−1^. The maximum anti-biofilm formation was seen for compound 7 at 300 μg mL^−1^ and an acceptable 70% cell viability. Since the amounts of compounds should be reduced when using in clinical (even as a single or combination treatment), we envision that the toxicity of compounds toward humans will be reduced. This data should be considered when developing the next generation of compounds, which is ongoing in our lab.

**Fig. 7:**
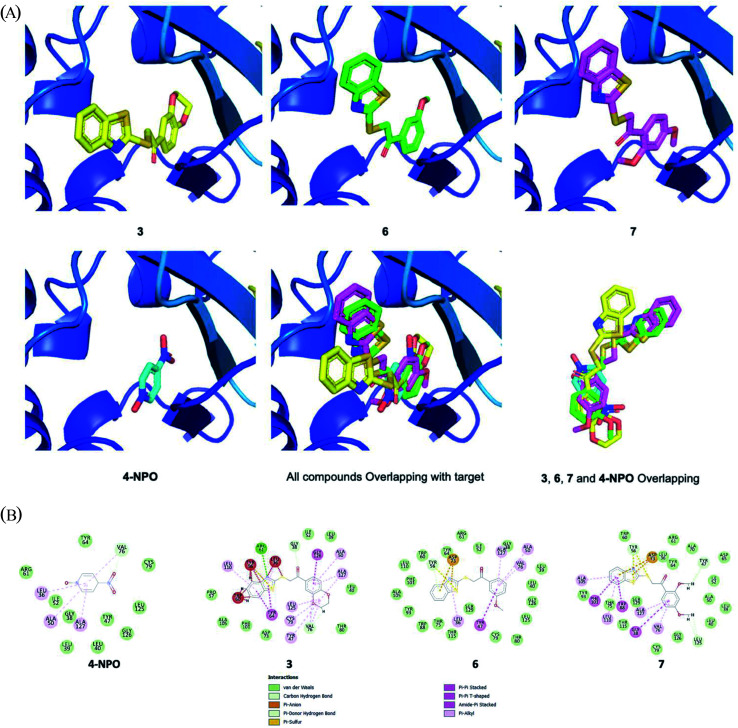
(A) Docking results of docking studies were performed for 3, 6, 7 and 4-NPO; (LasR ligand, PDB ID: 2UV0)). (B) Interaction of docked compounds with binding pocket.

Next, in order to initially understand the mechanism of action, docking studies were performed for 3, 6, 7 and reference compound 4-NPO. *Pseudomonas aeruginosa* quorum sensing *LasR* ligand (PDB ID: 2UV0) was selected as the target. The docking result is depicted in Fig. 7A. As a result, all compounds located perfectly to the active site of *Pseudomonas aeruginosa* quorum sensing LasR with a similar full-fitness score of around −3000 kcal mol^−1^ (Fig. 7A, Table 3). Compound 3, 6 and 7 had the same binding energy of around −8.80 kcal mol^−1^. In comparison to reference compound 4-NPO (docking score of −6.00 kcal mol^−1^), three hit compounds showed better docking scores (Table 3). Compounds 6 (green) and 7 (pink) showed very high overlapping figure (Fig. 7A), which suggests the same binding motif of interacting with binding protein pocket.

**Table 3:** Prediction of binding and fullfitness energy

Compound	Binding energy^a^ (kcal mol^−1^)	Fullfitness^a^ (kcal mol^−1^)
3	−9.60	−3003.51
	−8.70	−2995.28
	−7.76	−2983.76
6	−8.96	−3013.88
	−8.82	−3014.97
	−8.41	−3006.36
7	−8.82	−2998.77
	−8.02	−2988.95
	−7.90	−2983.57
4-NPO	−6.00	−3001.90
	−5.92	−3001.37
	−5.88	−3001.38

a Three best energy.

As depicted in Fig. 7B, several strong bindings of benzothiazole moiety of compounds 3, 6, 7 with protein pocket were observed. An interesting Pi–sulfur interaction of ASP-73 was seen for benzothiazole in 6 and 7. In terms of 3, the strong interaction of TRP-60, TYR-56, LEU-36 to the benzothiazole moiety stabilized the complex which resulted in best binding energy. Overall, the docking experiments reveal that the presence of benzothiazole moiety contribute to the QS activities of our compounds.

The difference between experimental results (where 4-NPO is strongest in QSI activity) could be explained by the difference of compounds in the physicochemical properties. Therefore, to better understand this mechanism, computational calculations of physicochemical properties of the compounds were performed in the next step.

**Fig. 8:**
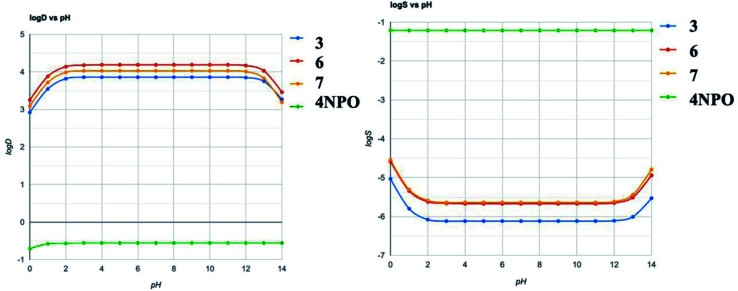
Computationally predicted pH-dependent log *S* and log *D* curve by chemAxon.

The calculations of drug-likeness of our hit compounds and ADME predictions including lipophilicity, physicochemical properties and pharmacokinetics were summarized in Table 4 and Fig. 8.

**Table 4:** ADME prediction of the hit compounds 3, 6, 7 and 4-NPO

Cpd	Formula	MW	#Rot^a^	#H-a^b^	#H-d^c^	MR^d^	TPSA^e^	Solubility^f^ (mg mL^−1^)	Sol. class^g^	GI abs.^h^	Bio.^i^	*P*-gp subs.^j^
3	C_17_H_13_NO_3_S_2_	343.42	4	4	0	91.93	101.96	4.05 × 10^−3^	Moderately soluble	High	0.55	No
6	C_16_H_13_NO_2_S_2_	315.41	5	3	0	87.55	92.73	4.48 × 10^−3^	Moderately soluble	High	0.55	No
7	C_17_H_15_NO_3_S_2_	345.44	6	4	0	94.04	101.96	3.84 × 10^−3^	Moderately soluble	High	0.55	No
4-NPO	C_5_H_4_N_2_O_3_	140.10	1	3	0	36.43	71.28	2.55 × 10^1^	Very soluble	High	0.55	No

a Number of rotation bond.

b Number of H-bond acceptors.

c Number of H-bond donors.

d Molecular refractivity.

e Topological polar surface area.

f Intrinsic solubility at 25 °C calculated by ESOL equation of Delaney.

g Solubility class, ESOL class.

h Gastrointestinal absorption: according to the white of the BOILED-Egg, SwissADME.

i Abbott bioavailability score: probability of *F* > 10% in rat calculated by SwissADME.

j *P*-Glycoprotein substrate: SVM model built on 1033 molecules (training set) and tested on 415 molecules (test set) 10-fold CV: ACC = 0.72/AUC = 0.77 external: ACC = 0.88/AUC = 0.94.

The ADME calculations of 3, 6, and 7 in comparison to 4-NPO were presented in Table 4. For detail, 3, 6, and 7 showed the similarities in physicochemical properties and pharmacokinetics. All compounds meet the criteria of drug-likeness: Lipinski rules. In comparison to 4-NPO, the most remarkable differences were molecular refractivity and solubilities. The low solubility of the synthesized compounds may be one of the factors for less active in experimental screening compared to 4-NPO. The calculated predicted pH-dependent log *S* and log *D* were depicted in Fig. 8.

The pH-dependent of solubility and distribution coefficient (log *S* and log *D*) prediction were depicted in Fig. 8. The results reveal that the reference compound 4-NPO and three hit compounds 3, 6, 7 were completely reversed in terms of solubility and distribution coefficient. In combination with ADME prediction, our calculations suggest that the physicochemical properties of the hit compounds could be one of the reasons for the low activity in QS compared to 4-NPO. Those calculations will be the primary parameters for the design of the next generation of benzo[*d*]thiazole-2-thiol bearing 2-oxo-2- substituted-phenylethan-1-yl series.

## Conclusion

In this work, we have synthesized and evaluated fifteen novel benzo[*d*]thiazole/quinoline-2-thiol bearing 2-oxo-2-substituted-phenylethan-1-yl compounds toward *Pseudomonas aeruginosa* for antimicrobial activities. Twelve compounds 1–2, 4–5, 8–15 show antimicrobial activities with MIC ranges of less than 64 μg mL^−1^ (14), 128 μg mL^−1^ (1, 5, 8, 9, 11, 12, 15), and 256 μg mL^−1^ (2, 4, 10, 13). In the series of benzo[*d*]thiazole, compounds 3, 6 and 7 exhibited promising QS inhibitors (IC_50_ of 115.2 μg mL^−1^ and 182.2 μg mL^−1^, and 45.5 μg mL^−1^, respectively) without affecting the growth of the bacteria under concentration tested. Especially, compound 7 showed clearly anti-biofilm formation with the ability to reduce of 70% biofilm biomass. Docking studies reveal that compounds had a good binding energy on the QS *LasR* system. In addition, under our docking model, the presence of benzothiazole moiety seems to contribute to the QS activities of the compounds. In terms of biology, noteworthy, compounds 3, 6, 7 selectivity inhibit the *LasB* over the *PqsR QS* system. Based on those results, the inhibition of other bacterial virulence factors for hit compounds 3, 6 and 7 will be performed which are ongoing in our lab. The results of intensive studies on QS system as well as the next generation of compounds will be reported in due course.

## Experimental section

### Materials and methods

#### Chemistry

Solvent, reagents were purchased from Sigma-Aldrich, TCI and were used as is. The reported NMR-spectra (^1^H-NMR, COSY, HMBC, HSQC) were recorded with 400/500 MHz Bruker Avance and the samples ran at 300 K. ^13^C-NMR were analyzed at 151 MHz or 101 MHz (as indicated). Chemical shifts (*δ*) are reported in ppm relative to the residual solvent peak as the internal standard. *J* values are reported in Hertz. Silica gel 60 F_254_ plates (pre-coated) were used for TLC and visualized under UV light. MP70 Mettler Toledo was used for melting points. All synthesized compounds possess at least 95% purity before biological studies. Purities were analyzed on a Waters 2795 system equipped with a Waters 996 PDA detector and a Waters Symmetry C18 Column (2.1 × 50 mm, 3.5 l m), flow rate 0.2 mL min^−1^. HRMS data were recorded on an electrospray (ESI) mass spectrometer. Microwave reactions were performed in an automatic Biotage® Initiator (robot sixty, an automated 60-position system). Microwave vials and aluminum seals are high precision standards.

#### Biology

##### Antimicrobial activities

Standard laboratory test strain *Pseudomonas aeruginosa* ATCC27853 (purchased from ATCC, Manassas, VA, USA) was used for antimicrobial activity experiments. Minimum inhibitory concentrations (MIC) ranges were determined as previously described (broth microdilution).^15^

##### Quorum sensing assay

Using the previously described methodology with a slightly modification.^16^*P. aeruginosa* quorum sensing reporter strains *lasB-gfp* was used for screening^17^ the inhibition of QS system. Culture of each reporter strain was grown for 20 h at 37 °C (shaking at 180 rpm). Then the overnight cultures were diluted to a final OD_450_ of 0.1. The assays were performed in 96-well microtiter dishes (Black Isoplate, Waltham Massachusetts, USA). Hit compounds, 4-NPO, growth media and reporter strains OHHL [*N*-(3-oxohexanoyl)-l-homoserine lactone] were added to the microtiter dishes. Growth and green fluorescent protein (GFP) expression were monitored using Victor X4 multilabel plate reader (Waltham Massachusetts, USA). The assays were maintained at 34 °C and measuring every 15 min (over 20 h). GFP expression was recorded as fluorescence at an excitation wavelength of 485 nm and an emission wavelength of 535 nm.

##### Docking studies

AutoDock Vina was used to perform the docking studies.^18^ The *Pseudomonas aeruginosa* quorum sensing *LasR* ligand (PDB ID: 2UV0) was used as the target.^20*a*^ The residues in the *LasR* binding pocket were allowed maximum flexibility. The three best binding compounds (energy scoring) were selected. UCSF-Chimera, version 1.12 was used for visualizing.^20*b*^

##### Computational calculation

ADME prediction was calculated using ChemAxon and online calculation service SwissADME.^21^

##### Procedure for the preparation of benzo[*d*]thiazole-2-thiol^22^

A mixture of 2-chloroaniline (1 equiv.), dimethylacetamide (10 mL), *O*-isopropylxanthic acid potassium salt (PIX) (2 equiv.) was added to a MW vial. The mixture was MW-ed at 150 °C for 5 min. After that, the vial was cooled to room temperature, followed by adding 50 mL of cold water. The mixture was adjusted to pH 3 by HCl 1 N. The precipitate was filtered and washed with H_2_O ×3 times to afford benzo[*d*]thiazole-2-thiol without additional purification.

##### General procedure for the preparation of final compound benzo[*d*]thiazole/quinoline-2-thiol bearing 2-oxo-2-substituted-phenylethan-1-yl

A mixture of 1-(substituted phenyl)ethan-1-one (1 eq.), copper(ii) bromide (1 eq.), 10 mL of mixture of 1 : 1 (v/v) of ethyl acetate and dichloromethane was added to a to a MW vial. The mixture was MW-ed at 60 °C, high absorption for 15 min. After the completion of reaction by TLC, 30 mL of water was added. The mixture was then extracted with ethyl acetate (3 × 100 mL). The organic layer was evaporated under low pressure to afford crude intermediates which is used in the next step without purification.

The crude product above was dissolved in 20 mL of acetonitrile and the thiol-substituted heterocycle (quinoline-2-thiol or benzo[*d*]thiazole-2-thiol prepared above, 1 eq.) was added and stirred at room temperature for 30 min until the appearance of a precipitate solid. The precipitated was then filtered and fast washed 3 × 10 mL with each acetonitrile, ethanol, hot acetone then recrystallization in ethanol to afford clean compounds 1–15.

#### Characterization of the final compounds

##### 2-(Benzo[*d*]thiazol-2-ylthio)-1-phenylethan-1-one (1)

Yield 89%; off-white solid; mp: 179.1–179.9 °C; ^1^H NMR (400 MHz, DMSO-*d*_6_) *δ* 8.09 (d, *J* = 7.9 Hz, 2H), 8.01 (d, *J* = 7.8 Hz, 1H), 7.75 (d, *J* = 8.0 Hz, 1H, H19), 7.71 (t, *J* = 7.5 Hz, 1H, H9), 7.57 (t, *J* = 7.4 Hz, 2H), 7.43 (t, *J* = 7.3 Hz, 1H), 7.36 (t, *J* = 7.5 Hz, 1H), 5.18 (s, 2H, CH_2_); ^13^C NMR (101 MHz, DMSO-*d*_6_) *δ* 193.41, 166.31, 152.89, 135.90, 135.24, 134.20, 129.33, 128.90, 126.79, 124.90, 122.32, 121.51, 41.53; LRMS(ESI) *m*/*z* [M + H]^+^ found 286.0; HRMS (ESI) *m*/*z* [M + H]^+^, calcd for C_15_H_12_NOS_2_^+^, 286.0360, found 316.0360.

##### 2-(Benzo[*d*]thiazol-2-ylthio)-1-(4-methoxyphenyl)ethan-1-one (2)

Yield 85%; off-white solid; mp: 195–196 °C; ^1^H NMR (600 MHz, DMSO-*d*_6_) *δ* 8.05 (d, *J* = 8.0 Hz, 2H), 7.98 (d, *J* = 8.1 Hz, 1H), 7.76 (d, *J* = 7.9 Hz), 7.43 (t, *J* = 7.8 Hz), 7.34 (t, *J* = 7.6 Hz), 7.09 (d, *J* = 8.6 Hz, 2H), 5.10 (s, 2H), 3.86 (s, 3H); ^13^C NMR (151 MHz, DMSO-*d*_6_) *δ* 191.64, 166.50, 164.11, 152.89, 135.24, 131.42, 128.60, 126.84, 124.92, 122.33, 121.50, 114.60, 56.11, 41.20; LRMS(ESI) *m*/*z* [M + H]^+^ found 316.0; HRMS (ESI) *m*/*z* [M + H]^+^, calcd for C_16_H_14_NO_2_S_2_^+^, 316.0468, found 316.0467.

##### 2-(Benzo[*d*]thiazol-2-ylthio)-1-(2,3-dihydrobenzo[*b*][1,4]dioxin-6-yl)ethan-1-one (3)

Yield 81%; white solid; mp: 160–162 °C; ^1^H NMR (400 MHz, DMSO-*d*_6_) *δ* 8.02 (dd, *J* = 8.0 Hz, *J* = 1.2 Hz, 1H), 7.81 (dd, *J* = 8.2 Hz, *J* = 1.1 Hz, 1H), 7.66 (dd, *J* = 8.4 Hz, *J* = 2.1 Hz, 1H), 7.59 (d, *J* = 2.1 Hz, 1H), 7.44 (m, 1H), 7.35 (m, 1H), 7.04 (d, *J* = 8.4 Hz, 1H), 5.08 (s, 2H), 4.30–4.37 (m, 4H); ^13^C NMR (101 MHz, DMSO-d6) *δ* 191.4(C=O), 166.30, 152.81, 149.01, 143.73, 135.23, 129.20, 126.79, 124.90, 123.10, 122.21, 121.54, 117.81, 117.71, 65.10, 64.43, 37.41; LRMS(ESI) *m*/*z* [M + H]^+^ found 344.0; HRMS (ESI) *m*/*z* [M + H]^+^, calcd for C_17_H_14_NO_3_S_2_^+^, 344.0415, found 344.0415.

##### 2-(Benzo[*d*]thiazol-2-ylthio)-1-(2-methoxyphenyl)ethan-1-one (4)

Yield 86%; colorless solid; mp: 170–172 °C; ^1^H NMR (600 MHz, DMSO-*d*_6_) *δ* 8.42 (br, 1H), 8.27 (br, 1H), 7.98 (dd, *J* = 8.0 Hz, *J* = 1.1 Hz), 7.74 (dd, *J* = 8.2 Hz, *J* = 1.0 Hz, 1H), 7.66 (dd, *J* = 7.7 Hz, *J* = 1.8 Hz), 7.60 (m, 1H), 7.43 (m, 1H), 7.32 (m, 1H), 7.21 (d, *J* = 8.4 Hz, 1H), 7.08 (m, 1H), 4.91 (s, 2H), 3.93 (s, 3H); ^13^C NMR (151 MHz, DMSO-*d*_6_) *δ* 194.22, 166.64, 159.21, 152.93, 135.26, 135.12, 130.62, 126.79, 126.01, 124.90, 122.22, 121.41, 121.11, 113.14, 117.70, 56.60, 45.32; LRMS(ESI) *m*/*z* [M + H]^+^ found 316.0; HRMS (ESI) *m*/*z* [M + H]^+^, calcd for C_16_H_14_NO_2_S_2_^+^, 316.0468, found 316.0467.

##### 2-(Benzo[*d*]thiazol-2-ylthio)-1-(3,4-dichlorophenyl)ethan-1-one (5)

Yield 89%; white solid; mp: 186–187 °C; ^1^H NMR (600 MHz, DMSO-*d*_6_) *δ* 8.34 (br, 1H), 8.07 (br, 2H), 7.86 (d, *J* = 8.4 Hz, 1H), 7.77 (d, *J* = 8.1 Hz, 1H), 7.45 (t, 1H, *J* = 7.4 Hz), 7.35 (t, *J* = 7.7 Hz, 1H), 5.16 (s, 2H); ^13^C NMR (151 MHz, DMSO-*d*_6_) *δ* 191.92, 165.94, 152.81, 137.11, 136.17, 135.34, 132.44, 131.76, 130.92, 128.92, 126.83, 125.05, 122.31, 121.56, 41.22; LRMS(ESI) *m*/*z* [M + H]^+^ found 353.9; HRMS (ESI) *m*/*z* [M + H]^+^, calcd for C_15_H_10_Cl_2_NOS_2_^+^, 353.9580, found 353.9581.

##### 2-(Benzo[*d*]thiazol-2-ylthio)-1-(3-methoxyphenyl)ethan-1-one (6)

Yield 89%; yellow solid; mp: 170–171 °C; ^1^H NMR (600 MHz, DMSO-*d*_6_) *δ* 8.01 (d, *J* = 8.0 Hz, 1H), 7.76 (d, *J* = 8.1 Hz, 1H), 7.70 (d, *J* = 7.7 Hz, 1H), 7.57 (d, *J* = 2.4 Hz, 1H), 7.51 (t, *J* = 7.9 Hz, 1H), 7.45 (t, *J* = 7.6 Hz, 1H), 7.35 (t, *J* = 7.6 Hz, 1H), 7.29 (dd, *J* = 8.3 Hz, *J* = 2.5 Hz), 5.17 (s, 2H), 3.83 (s, 3H); ^13^C NMR (151 MHz, DMSO-*d*_6_) *δ* 193.24, 166.31, 159.98, 152.94, 137.25, 135.21, 130.50, 126.81, 124.96, 122.31, 121.55, 121.45, 120.39, 113.48, 55.93, 41.61; LRMS(ESI) *m*/*z* [M + H]^+^ found 316.0; HRMS (ESI) *m*/*z* [M + H]^+^, calcd for C_16_H_14_NO_2_S_2_^+^, 316.0468, found 316.0468.

##### 2-(Benzo[*d*]thiazol-2-ylthio)-1-(2,4-dimethoxyphenyl)ethan-1-one (7)

Yield 72%; yellow solid; mp: 207–208 °C; ^1^H NMR (600 MHz, DMSO-*d*_6_) *δ* 7.98 (dd, *J* = 1.3 Hz, *J* = 0.5 Hz, 1H), 7.77 (d, *J* = 5.7 Hz, 1H), 7.73 (d, *J* = 8.8 Hz, 1H), 7.41 (m, 1H), 7.31 (m, 1H), 6.70 (d, *J* = 2.2 Hz, 1H), 6.66 (dd, *J* = 8.8 Hz, *J* = 2.3 Hz, 1H), 4.88 (s, 2H), 3.96 (s, 3H, H23), 3.85 (s, 3H); ^13^C NMR (151 MHz, DMSO-*d*_6_) *δ* 190.85, 166.32, 164.93, 160.91, 152.45, 134.44, 132.28, 126.11, 124.12, 121.56, 120.81, 117.85, 106.57, 98.20, 56.01, 55.61, 45.03; LRMS(ESI) *m*/*z* [M + H]^+^ found 346.0; HRMS (ESI) *m*/*z* [M + H]^+^, calcd for C_17_H_16_NO_3_S_2_^+^, 346.0572, found 346.0573.

##### 1-(2-Methoxyphenyl)-2-(quinolin-2-ylthio)ethan-1-one (8)

Yield 82%; off-white solid; mp: 204.0–204.5 °C; ^1^H NMR (600 MHz, DMSO-*d*_6_) *δ* 8.18 (d, *J* = 8.6 Hz, 1H), 7.93 (d, *J* = 6.7 Hz, 1H), 7.65 (dtd, *J* = 6.9 Hz, *J* = 1.5 Hz, 1H), 7.59 (m, 3H), 7.47 (dtd, *J* = 2.5 Hz, *J* = 1.3 Hz, 1H), 7.45 (d, *J* = 8.7 Hz, 1H), 7.25 (d, *J* = 8.2 Hz, 1H), 7.06 (td, *J* = 6.5 Hz, *J* = 0.95 Hz, 1H), 4.74 (s, 2H), 3.95 (s, 3H); ^13^C NMR (151 MHz, DMSO-*d*_6_) *δ* 196.33, 158.67, 158.40, 147.64, 136.62, 134.41, 130.55, 130.43, 128.55, 127.73, 127.48, 126.19, 125.90, 120.91, 120.82, 112.96, 56.47, 41.42; LRMS(ESI) *m*/*z* [M + H]^+^ found 310.1; HRMS (ESI) *m*/*z* [M + H]^+^, calcd for C_18_H_16_NO_2_S^+^, 310.0902, found 310.0903.

##### 1-(2,3-Dihydrobenzo[*b*][1,4]dioxin-6-yl)-2-(quinolin-2-ylthio)ethan-1-one (9)

Yield 85%; yellow solid; mp: 227–228 °C; ^1^H NMR (600 MHz, DMSO-*d*_6_) *δ* 7.92 (d, *J* = 8.7 Hz, 1H), 7.81 (d, *J* = 8.2 Hz, 1H), 7.71 (br, 2H, H1), 7.70 (d, *J* = 2.1 Hz, 1H), 7.61 (t, *J* = 7.0 Hz, 1H), 7.42 (t, *J* = 7.0 Hz, 1H), 7.28 (d, *J* = 8.6 Hz, 1H), 6.95 (d, *J* = 8.9 Hz, 1H), 4.81 (s, 2H), 4.33 (m, 2H), 4.29 (m, 2H); ^13^C NMR (151 MHz, DMSO-*d*_6_) *δ* 193.13, 157.48, 148.33, 147.86, 143.41, 135.90, 130.12, 129.81, 127.66, 127.65, 126.11, 125.54, 122.93, 120.64, 118.20, 117.31, 64.78, 64.12, 36.62; LRMS(ESI) *m*/*z* [M + H]^+^ found 338.0; HRMS (ESI) *m*/*z* [M + H]^+^, calcd for C_19_H_16_NO_3_S^+^, 338.0851, found 338.0853.

##### 1-(3-Methoxyphenyl)-2-(quinolin-2-ylthio)ethan-1-one (10)

Yield 73%; white solid; mp: 220–221 °C; ^1^H NMR (600 MHz, DMSO-*d*_6_) *δ* 8.24 (d, *J* = 8.1 Hz, 1H), 7.93 (d, *J* = 6.6 Hz, 1H), 7.78 (d, *J* = 7.7 Hz, 1H, H16), 7.67 (dtd, *J* = 6.8H, *J* = 1.5 Hz, 1H), 7.60 (q, *J* = 1.6 Hz, *J* = 1.1 Hz, *J* = 1.6 Hz), 7.46–7.56 (m, 4H), 7.29–7.33 (dq, *J* = 7.7 Hz, *J* = 0.94 Hz, 1H), 4.90 (s, 2H), 3.82 (s, 3H); ^13^C NMR (151 MHz, DMSO-*d*_6_) *δ* 194.71, 159.74, 158.07, 147.63, 138.33, 136.90, 130.62, 130.41, 128.55, 127.18, 126.10, 125.99, 121.35, 120.80, 119.81, 112.31, 55.93, 37.32; LRMS(ESI) *m*/*z* [M + H]^+^ found 310.1; HRMS (ESI) *m*/*z* [M + H]^+^, calcd for C_18_H_16_NO_2_S^+^, 310.0902, found 310.0902.

##### 1-(4-Hydroxy-3-methoxyphenyl)-2-(quinolin-2-ylthio)ethan-1-one (11)

Yield 95%; yellow solid; mp: 243–245 °C; ^1^H NMR (600 MHz, DMSO-*d*_6_) *δ* 8.20 (d, *J* = 8.7 Hz, 1H), 7.89 (d, *J* = 8.0 Hz, 1H), 7.73 (d, *J* = 8.3 Hz, 1H), 7.66 (t, *J* = 7.8 Hz, 1H), 7.62 (d, *J* = 8.7 Hz, 1H, H3), 7.54 (s, 1H), 7.44–7.49 (br, 2H, H1), 6.92 (d, *J* = 8.3 Hz, 1H), 4.85 (s, 2H), 3.78 (s, 3H); ^13^C NMR (151 MHz, DMSO-*d*_6_) *δ* 192.42, 157.90, 152.07, 147.51, 146.94, 136.50, 130.21, 128.06, 127.91, 126.73, 125.71, 125.60, 123.52, 120.43, 115.01, 111.60, 55.63, 36.22; LRMS(ESI) *m*/*z* [M + H]^+^ found 326.1; HRMS (ESI) *m*/*z* [M + H]^+^, calcd for C_18_H_16_NO_3_S^+^, 326.0851, found 326.0852.

##### 1-Phenyl-2-(quinolin-2-ylthio)ethan-1-one (12)

Yield 93%; off-white solid; mp: 188–189 °C; ^1^H NMR (600 MHz, DMSO-*d*_6_) *δ* 8.25 (d, *J* = 8.7 Hz, 1H), 8.15 (dt, *J* = 7.7 Hz, *J* = 1.4 Hz, 2H), 7.93 (dd, *J* = 8.0 Hz, *J* = 1.4 Hz, 1H), 7.72 (m, 1H), 7.65 (m, 1H), 7.61 (m, 2H), 7.52 (m, 3H), 4.95 (s, 2H); ^13^C NMR (151 MHz, DMSO-*d*_6_) *δ* 194.73, 158.31, 147.04, 137.21, 136.81, 133.84, 130.82, 129.21, 128.83, 128.56, 126.81, 126.23, 126.15, 120.83, 37.42; LRMS(ESI) *m*/*z* [M + H]^+^ found 280.1; HRMS (ESI) *m*/*z* [M + H]^+^, calcd for C_17_H_14_NO_2_S^+^, 280.0796, found 280.0795.

##### 1-(4-Methoxyphenyl)-2-(quinolin-2-ylthio)ethan-1-one (13)

Yield 91%; yellow solid; mp: 201–202 °C; ^1^H NMR (600 MHz, DMSO-*d*_6_) *δ* 8.21 (d, *J* = 8.7 Hz, 1H), 8.10 (dt, *J* = 8.3 Hz, *J* = 2.1 Hz, 2H), 7.89 (d, *J* = 1.4 Hz, 1H), 7.65 (dtd, *J* = 6.7 Hz, *J* = 1.5 Hz, 1H), 7.57 (d, *J* = 7.6 Hz, 1H), 7.46–7.49 (br, 2H), 7.07–7.09 (dt, *J* = 7.9 Hz, *J* = 2.7 Hz, 2H), 4.89 (s, 2H), 3.86 (s, 3H); ^13^C NMR (151 MHz, DMSO-*d*_6_) *δ* 193.01, 163.82, 158.44, 147.14, 137.21, 131.27, 130.82, 129.54, 128.53, 126.93, 126.25, 126.11, 120.92, 114.46, 119.81, 112.31, 56.10, 37.10; LRMS(ESI) *m*/*z* [M + H]^+^ found 310.1; HRMS (ESI) *m*/*z* [M + H]^+^, calcd for C_18_H_16_NO_2_S^+^, 310.0902, found 310.0902.

##### 1-(4-Hydroxyphenyl)-2-(quinolin-2-ylthio)ethan-1-one (14)

Yield 89%; off-white solid; mp: 206–207 °C; ^1^H NMR (400 MHz, DMSO-*d*_6_) *δ* 8.28 (d, *J* = 8.7 Hz, 1H), 8.04 (dd, *J* = 8.8 Hz, *J* = 1.6 Hz, 2H), 7.94 (d, *J* = 8.3 Hz, 1H), 7.69 (m, 2H), 7.54 (m, 2H, H1, H9), 6.94 (d, *J* = 7.1 Hz), 4.92 (s, 2H); ^13^C NMR (101 MHz, DMSO-*d*_6_) *δ* 192.61, 162.80, 158.70, 146.54, 137.72, 131.51, 131.11, 128.62, 128.01, 126.41, 126.33, 126.28, 121.09, 115.81, 37.20; LRMS(ESI) *m*/*z* [M + H]^+^ found 296.0; HRMS (ESI) *m*/*z* [M + H]^+^, calcd for C_17_H_14_NO_2_S^+^, 296.0745, found 296.0746.

##### 1-(2,4-Dimethoxyphenyl)-2-(quinolin-2-ylthio)ethan-1-one (15)

Yield 91%; light yellow solid; mp: 192–194 °C; ^1^H NMR (600 MHz, DMSO-*d*_6_) *δ* 8.22 (d, *J* = 8.6 Hz, 1H), 7.91 (d, *J* = 8.1 Hz, 1H), 7.64–7.71 (m, 3H), 7.50 (t, *J* = 7.4 Hz, 1H), 7.45 (d, *J* = 8.6 Hz, 1H), 6.73 (br, 1H), 6.65 (dd, *J* = 8.7 Hz, *J* = 2.4 Hz, 1H), 4.78 (s, 2H), 3.97 (s, 3H), 3.87 (s, 3H); ^13^C NMR (151 MHz, DMSO-*d*_6_) *δ* 193.33, 165.018, 161.22, 158.90, 147.14, 137.10, 131.71, 130.82, 128.54, 126.93, 126.11, 126.04, 120.90, 119.84, 106.71, 98.90, 56.62, 56.24, 41.80; LRMS(ESI) *m*/*z* [M + H]^+^ found 340.0; HRMS (ESI) *m*/*z* [M + H]^+^, calcd for C_19_H_18_NO_3_S^+^, 340.1007, found 340.1008.

## Conflicts of interest

There are no conflicts to declare.

## Supplementary Material

RA-011-D1RA03616E-s001
