# Correction: Benzo[*d*]thiazole-2-thiol bearing 2-oxo-2-substituted-phenylethan-1-yl as potent selective *lasB* quorum sensing inhibitors of Gram-negative bacteria

**DOI:** 10.1039/d1ra90142g

**Published:** 2021-09-08

**Authors:** Tung Truong Thanh, Huy Luong Xuan, Thang Nguyen Quoc

**Affiliations:** Faculty of Pharmacy, PHENIKAA University Hanoi 12116 Vietnam tung.truongthanh@phenikaa-uni.edu.vn; PHENIKAA Institute for Advanced Study (PIAS), PHENIKAA University Hanoi 12116 Vietnam; Nuclear Medicine Unit, Vinmec Healthcare System Hanoi 10000 Vietnam

## Abstract

Correction for ‘Benzo[*d*]thiazole-2-thiol bearing 2-oxo-2-substituted-phenylethan-1-yl as potent selective *lasB* quorum sensing inhibitors of Gram-negative bacteria’ by Tung Truong Thanh *et al.*, *RSC Adv.*, 2021, **11**, 28797–28808, DOI: 10.1039/D1RA03616E.

The authors regret that the one of the affiliations (affiliation *a*) was incorrectly shown in the original manuscript. The corrected list of affiliations is as shown here.

The Royal Society of Chemistry apologises for these errors and any consequent inconvenience to authors and readers.

## Supplementary Material

