# Supplementary material for: Benzo[d]thiazole-2-thiol bearing 2-oxo-2-substituted-phenylethan-1-yl as potent selective lasB quorum sensing inhibitors of Gram-negative bacteria
Source: RSC Adv. 2021 Aug 26;11(46):28797–808. doi: 10.1039/d1ra03616e (PMC9038142; doi:10.1039/d1ra03616e)
Supplement: RA-011-D1RA03616E-s001 [file RA-011-D1RA03616E-s001.pdf]

Electronic Supplementary Material (ESI) for RSC Advances.
This journal is © The Royal Society of Chemistry 2021

Supporting information

For

Benzo[*d*]thiazole-2-thiol bearing 2-oxo-2-substituted-phenylethan-1-yl as potent selective *lasB* quorum sensing inhibitors of gram-negative bacteria.

Tung Truong Thanh,[1,2,] Huy Luong Xuan,[1,2] Thang Nguyen Quoc[3]*

[1]*Faculty of Pharmacy, PHENIKAA University, Hanoi 12116, Vietnam*
[2]*PHENIKAA Institute for Advanced Study (PIAS), PHENIKAA University, Hanoi 12116, Vietnam*
[3]*Nuclear Medicine Unit, Vinmec International Hospital, Hanoi 10000, Vietnam*

*Corresponding author
Email: tung.truongthanh@phenikaa-uni.edu.vn
ORCID: 0000-0002-5263-203X
tunglab.com

1. Cytotoxicity Assay.

The method was described previously [1]. Firstly, the stock solution of the active compounds **3, 6, 7** was prepared in DMSO as 200 ug/mL. The testing concentrations were prepared from stock solution by diluting in growth medium (90% high glucose medium supplemented with 10% fetal bovine serum). The HeLa cells were grown as monolayers in the growth medium at 37°C (atmosphere containing 5% CO_2). When cells reached 70% confluence were detached from the culture flask with 5% trypsin-EDTA and resuspended in fresh culture media at a density of 5 x 104 cells/mL. By use of a Falcon 24-well, flat bottom plate, 500 µL of the cell suspension was added to each of the wells, and the cells were incubated for 24h at 37°C. Then the active compounds (with concentrations of 1 to 200 µg/mL) were added to each cell in triplicates and incubated for 24 h. The cytotoxicity was performed using the in vitro toxicology assay kit, MTT based (Sigma). Absorbance values were measured at 570 nm and 690 nm. The absorbance values measured at 690 nm were subtracted from the values measured at 570 nm when the data was analyzed. Data were normalized by subtracting the absorbance values of the growth medium treated equally than the rest of the samples.

[1] Truong-Thanh Tung, Trong T Dao, Marta G Junyent, Michael Palmgren, Thomas Günther-Pomorski, Anja T Fuglsang, Søren B Christensen, John Nielsen, *ChemMedChem*, **2018**, 13(1):37-47

2. Copy of NMR spectra of library compounds

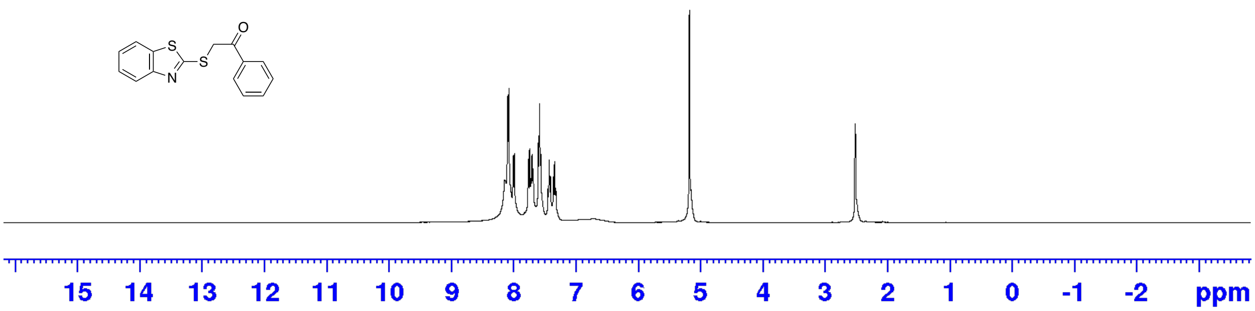

Figure S1. 1H of compound **1**

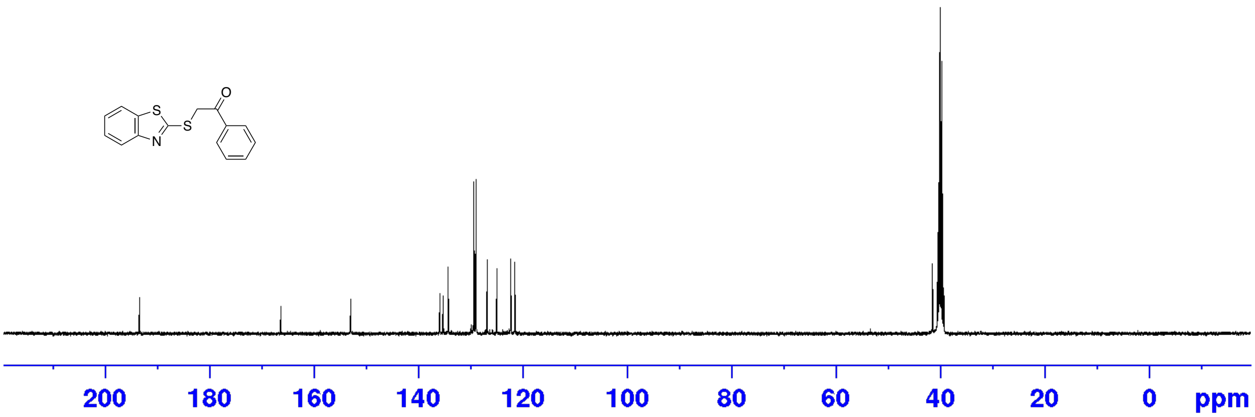

Figure S2. 13C of compound **1**

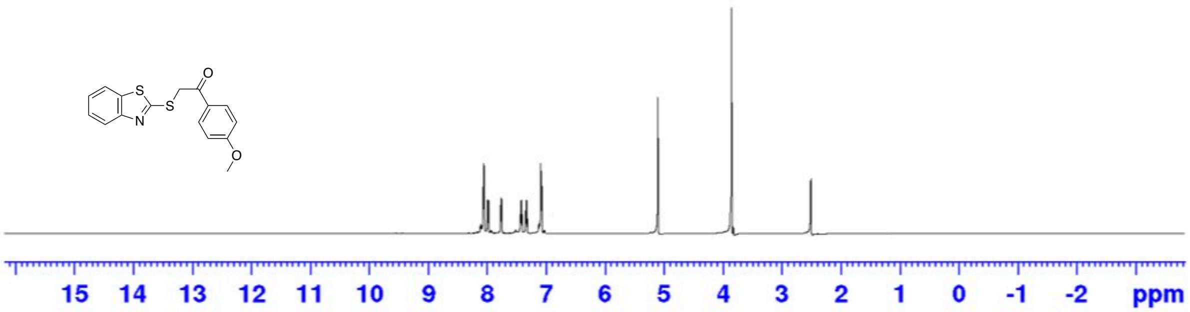

Figure S3. 1H of compound **2**

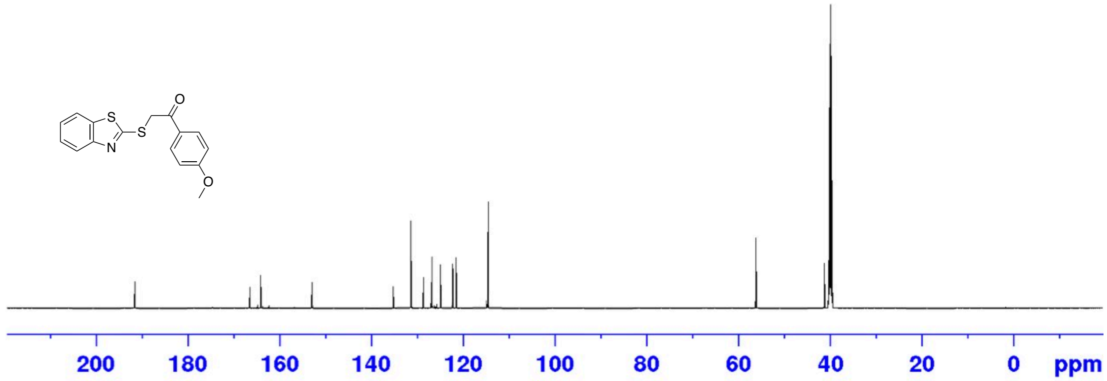

Figure S4. 13C of compound **2**

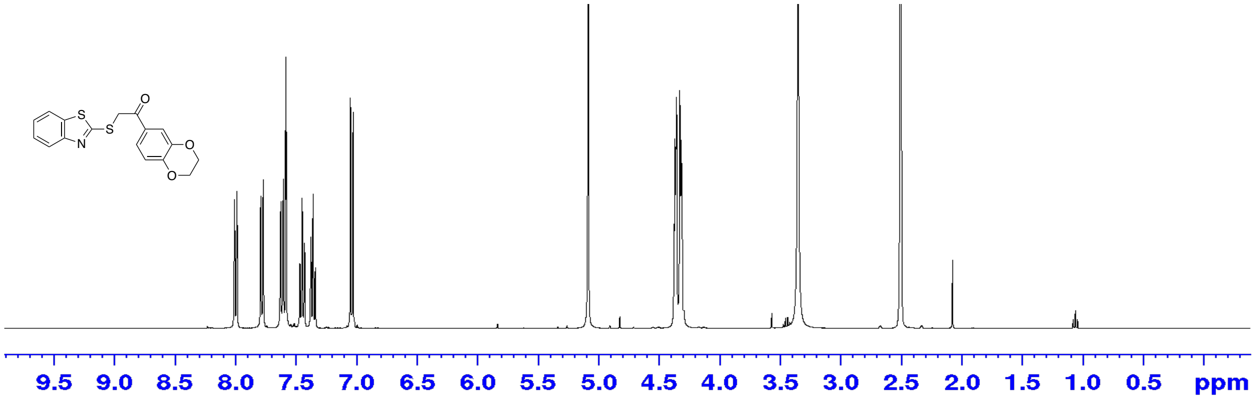

Figure S5. 1H of compound **3**

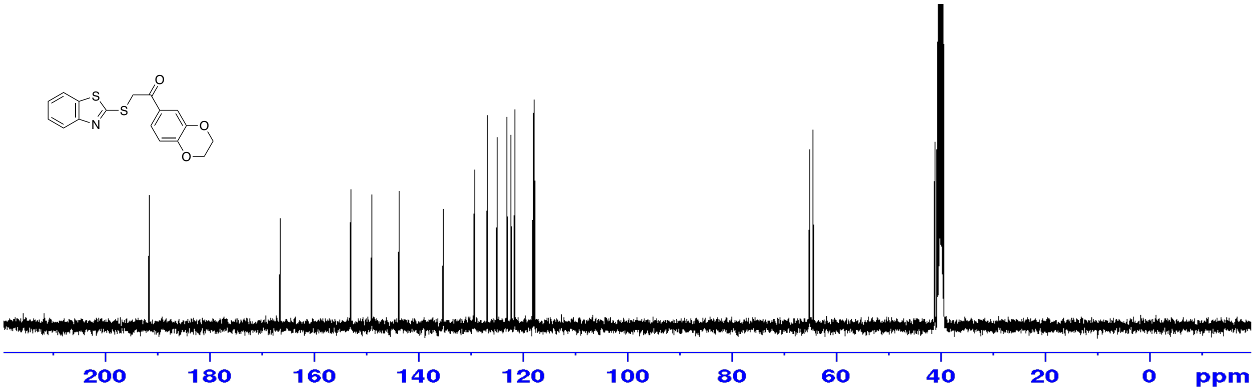

Figure S6. 13C of compound **3**

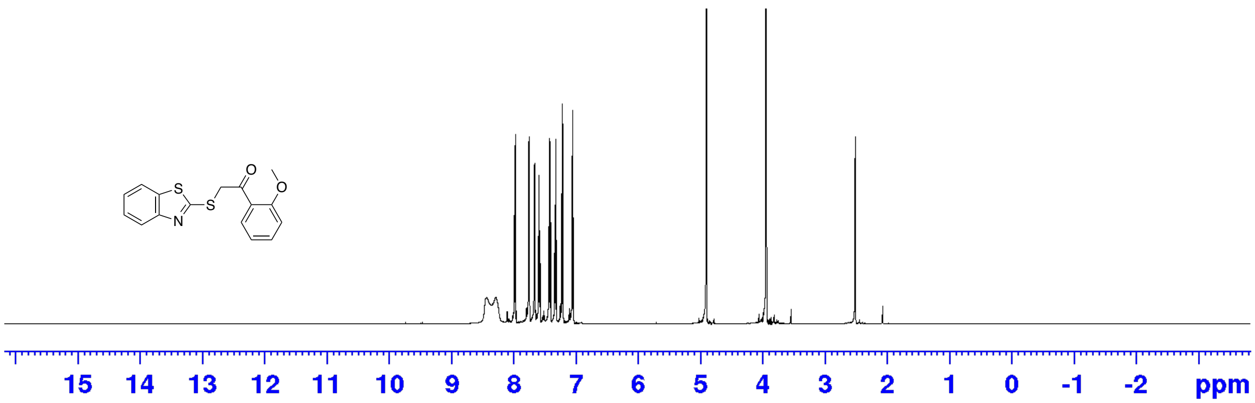

Figure S7. 1H of compound **4**

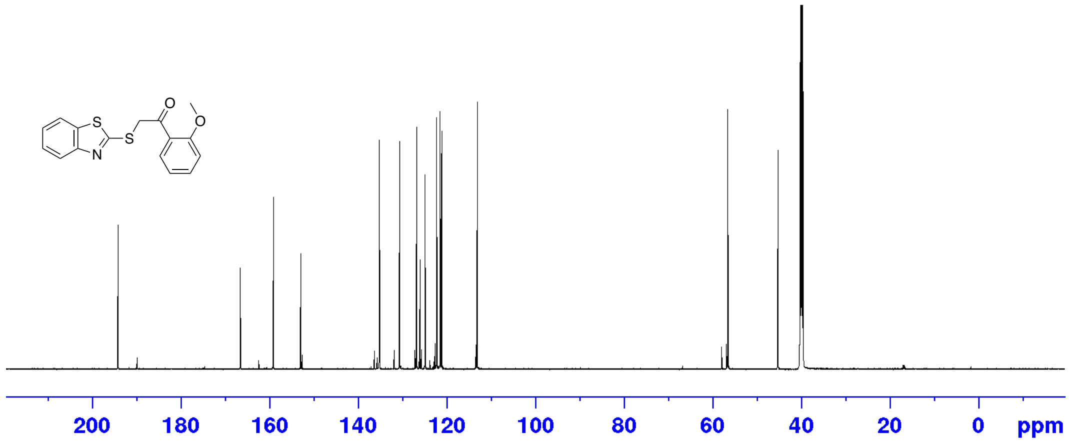

Figure S8. 13C of compound **4**

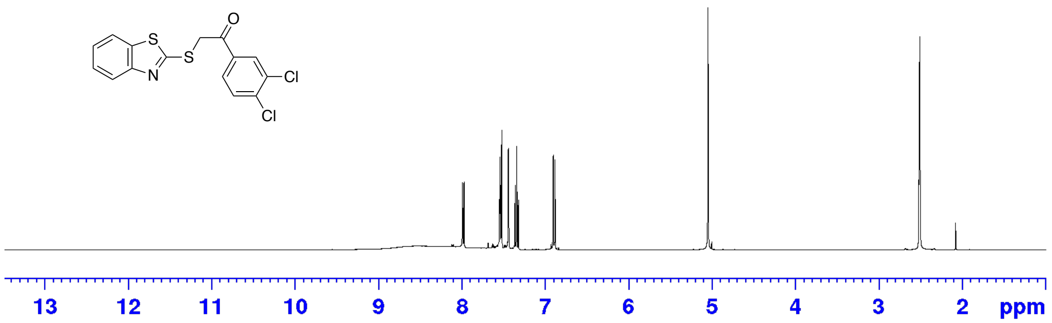

Figure S9. 1H of compound **5**

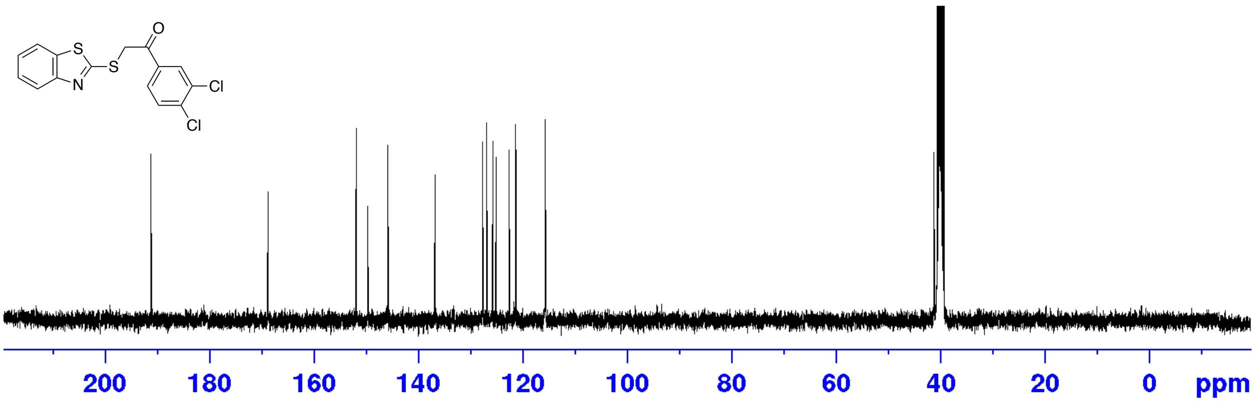

Figure S10. 13C of compound **5**

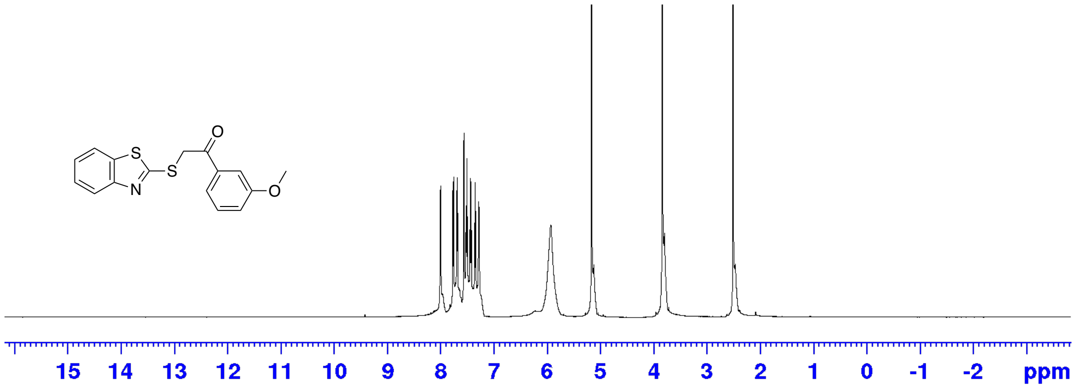

Figure S11. 1H of compound **6**

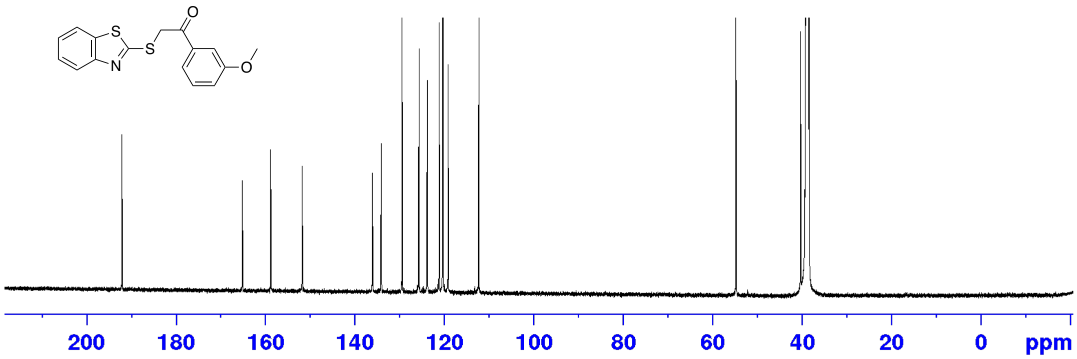

Figure S12. 13C of compound **6**

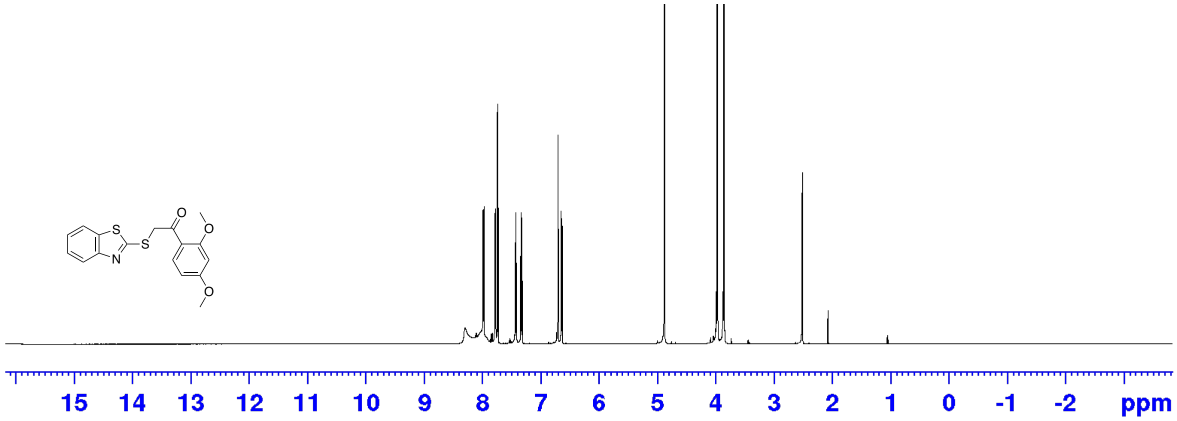

Figure S13. 1H of compound **7**

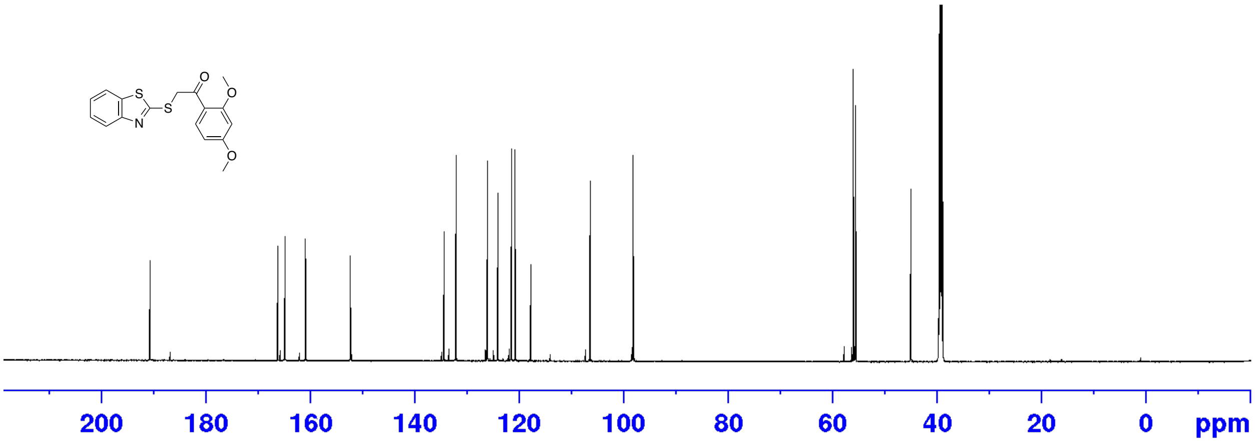

Figure S14. 13C of compound **7**

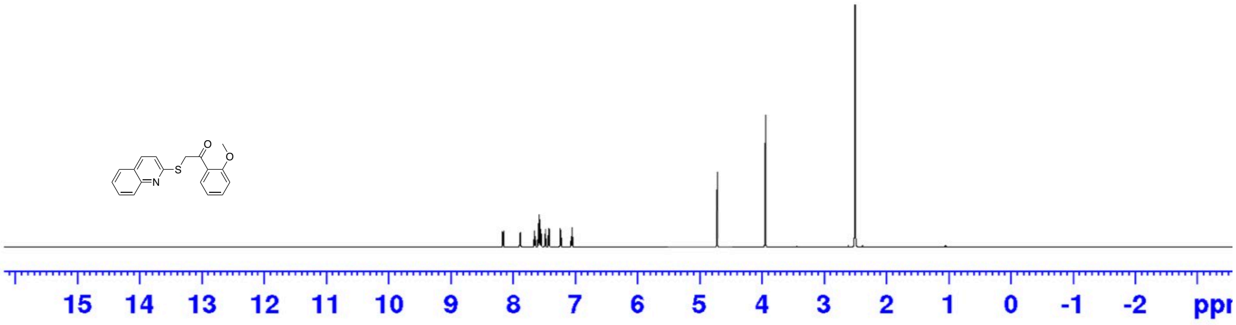

Figure S15. 1H of compound **8**

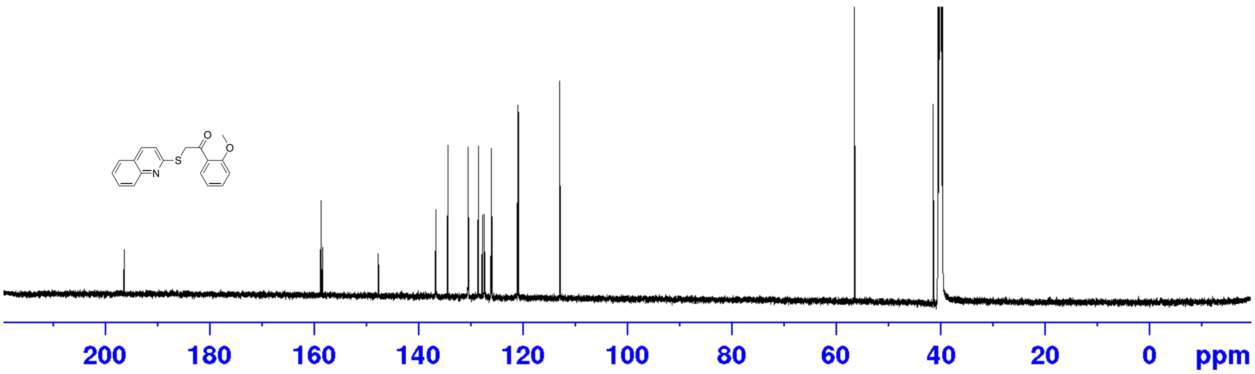

Figure S16. 13C of compound **8**

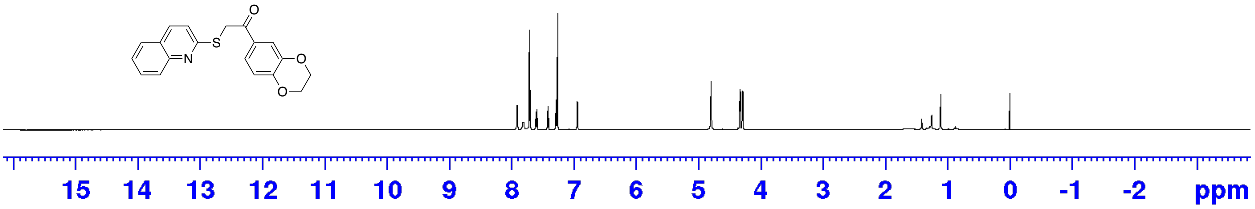

Figure S17. 1H of compound **9**

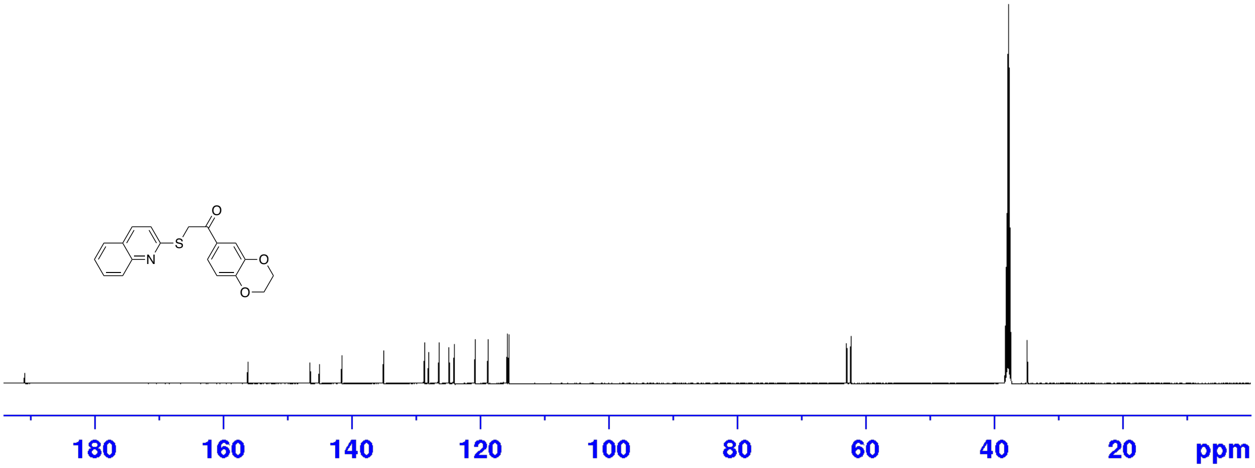

Figure S18. 13C of compound **9**

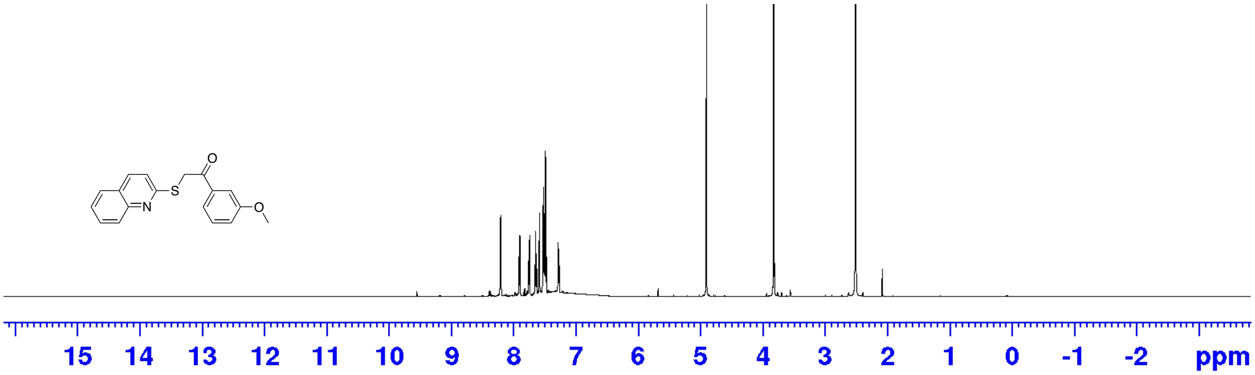

Figure S19. 1H of compound **10**

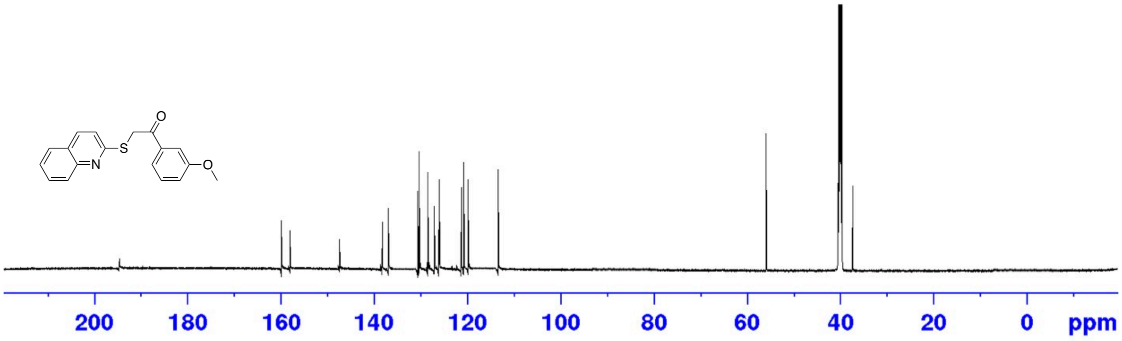

Figure S20. 13C of compound **10**

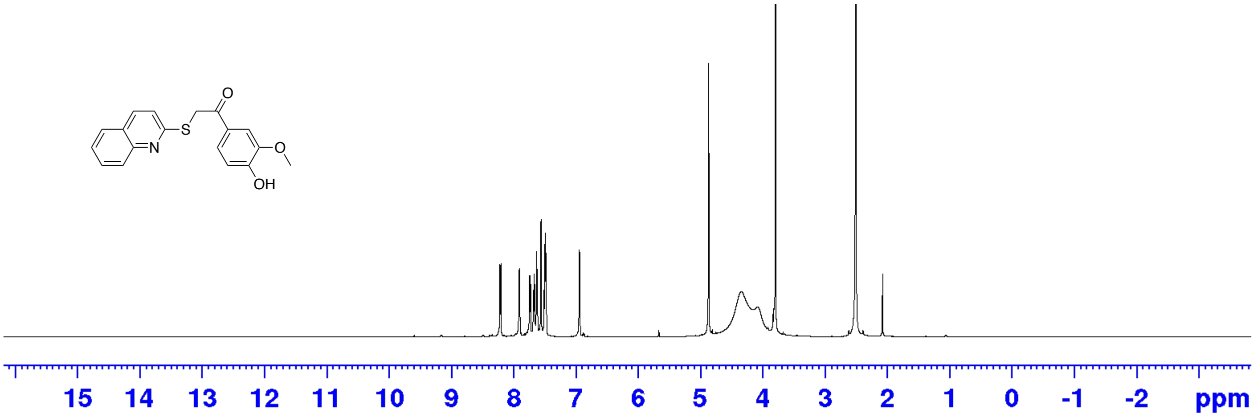

Figure S21. 1H of compound **11**

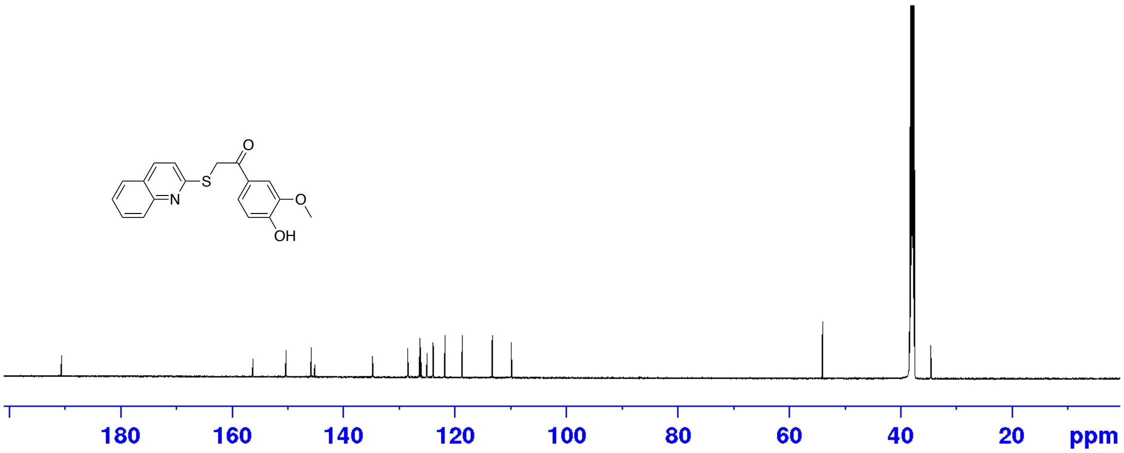

Figure S22. 13C of compound **11**

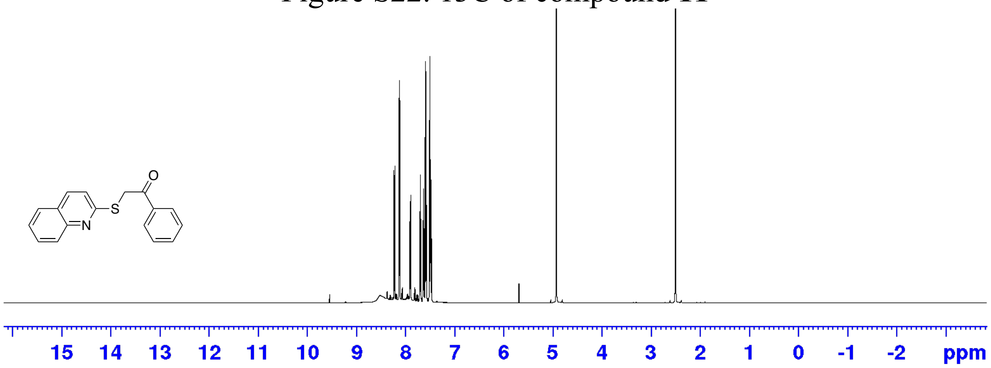

Figure S23. 1H of compound **12**

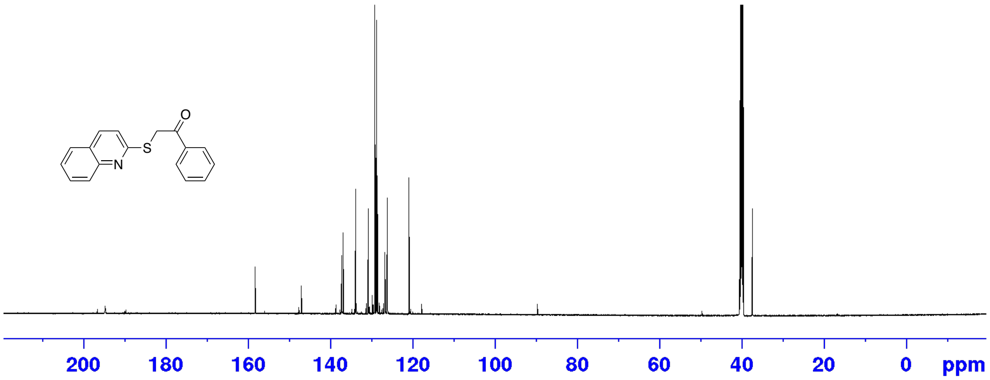

Figure S24. 13C of compound **12**

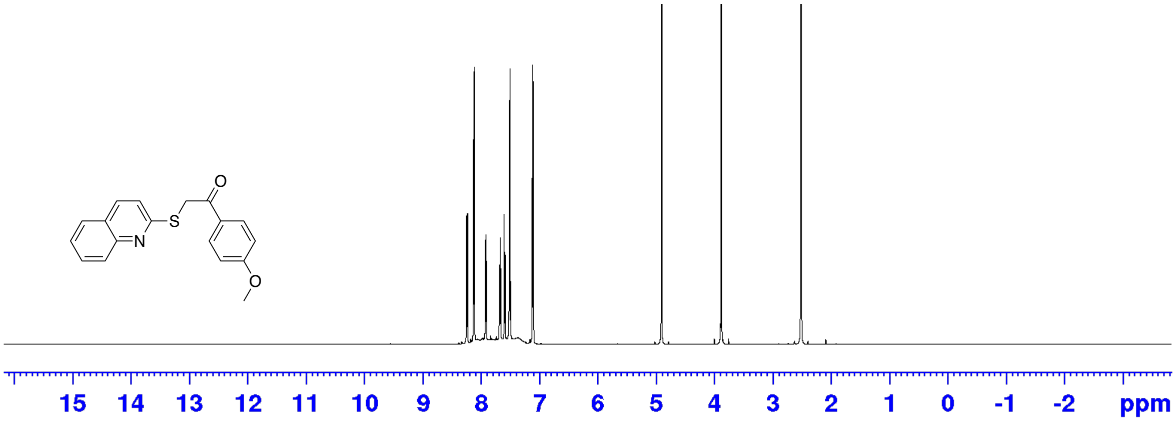

Figure S25. 1H of compound **13**

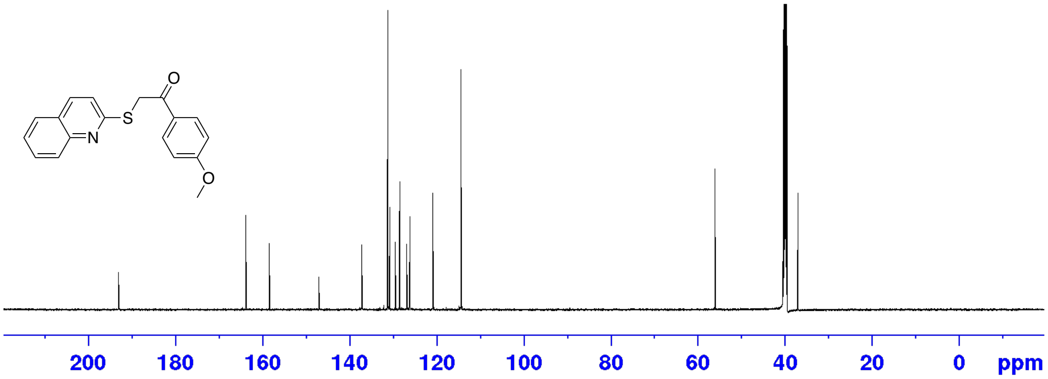

Figure S26. 13C of compound **13**

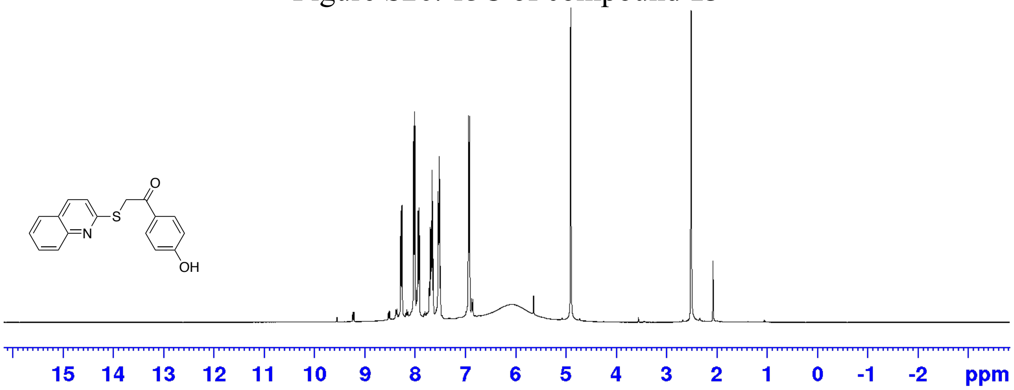

Figure S27. 1H of compound **14**

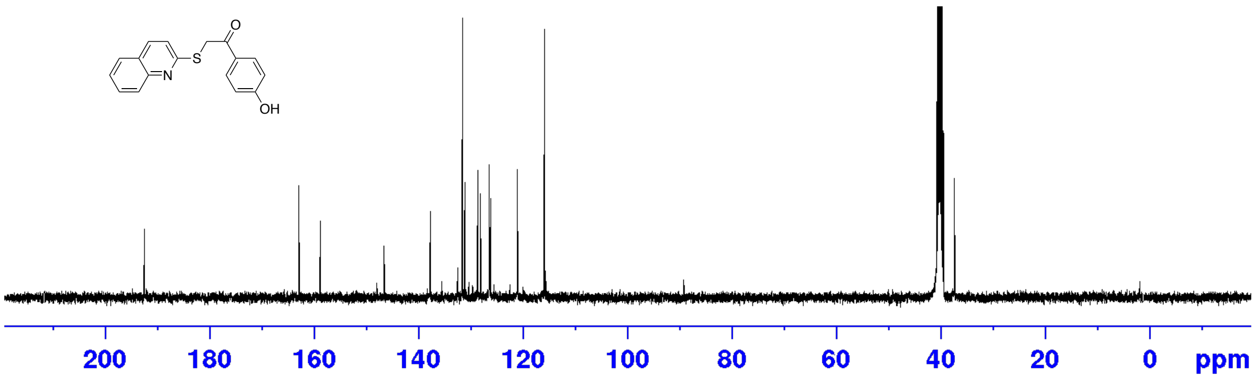

Figure S28. 13C of compound **14**

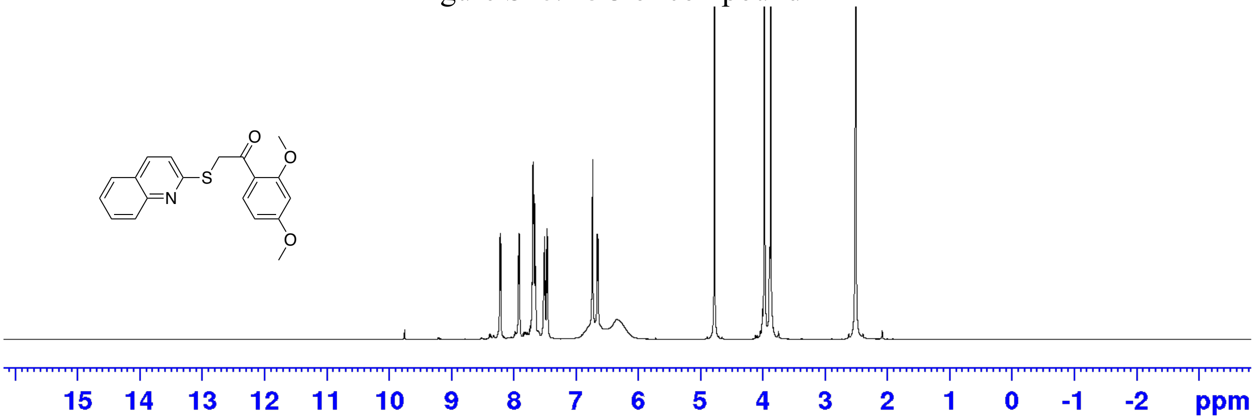

Figure S29. 1H of compound **15**

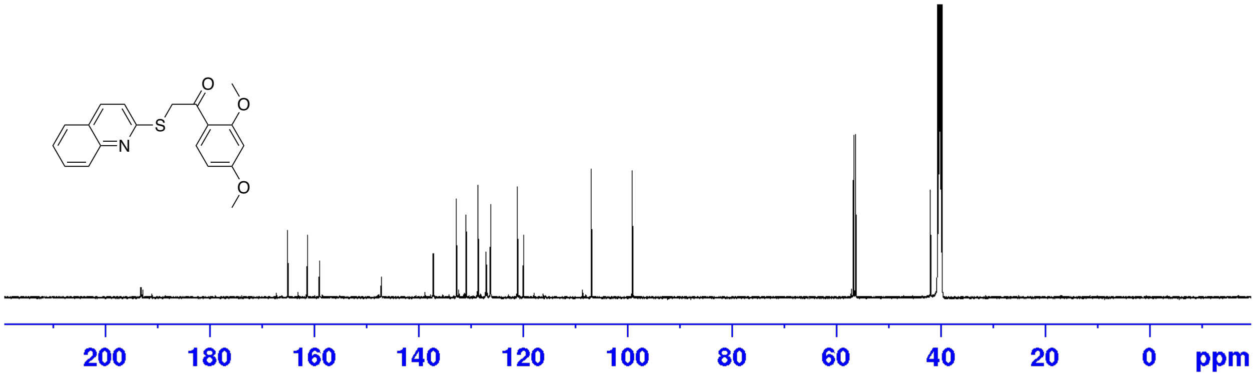

Figure S30. 1H of compound **15**
